# Effectiveness, safety, and patterns of use of camrelizumab in advanced esophageal cancer: an individual patient data pooled analysis of 987 patients from three prospective cohort studies

**DOI:** 10.1007/s00262-025-03970-z

**Published:** 2025-03-08

**Authors:** Zhihao Lu, Guoping Sun, Jiancheng Li, Jun Zhao, Zishu Wang, Dong Qian, Zhe Yang, Na Li, Junsheng Wang, Shuanghu Yuan, Yusheng Wang, Suyi Li, Zhen Yang, Fengming Ran, Yinghua Ji, Shaojin Zhu, Yanqiao Zhang, Chen Wang, Lixin Wan, Rongrong Zheng, Wenjie Deng, Fengzhuo Cheng, Lin Shen

**Affiliations:** 1 Department of Gastrointestinal Oncology, Peking University Cancer Hospital and Institute, 52 Fucheng Road, Beijing, China; 2 Department of Medical Oncology, The First Affiliated Hospital of Anhui Medical University, Hefei, China; 3 Department of Radiation Oncology, Fujian Cancer Hospital and Fujian Medical University Cancer Hospital, Fuzhou, China; 4 Department of Oncology, Changzhi People’s Hospital of Changzhi Medical College, Changzhi, China; 5 Department of Oncology, The First Affiliated Hospital of Bengbu Medical College, Bengbu, China; 6 Department of Radiation Oncology, The First Affiliated Hospital of USTC, Anhui Provincial Hospital, Hefei, China; 7 Cancer Research and Treatment Center, Shandong Provincial Hospital Affiliated to Shandong University, Jinan, China; 8 Department of Oncology, Suining Central Hospital, Suining, China; 9 Department of Internal Medicine, Anyang Cancer Hospital, Anyang, China; 10 Department of Radiation Oncology, Shandong Cancer Hospital and Institute, Jinan, China; 11 Department of Gastroenterology, The First Hospital of Shanxi Medical University, Taiyuan, China; 12 Department of Oncology, The First Affiliated Hospital of USTC, Anhui Provincial Hospital, Hefei, China; 13 Department of Oncology, The Second Affiliated Hospital of Anhui Medical University, Hefei, China; 14 Department of Oncology, Hubei Cancer Hospital, Tongji Medical College, Huazhong University of Science and Technology, Wuhan, China; 15 Department of Oncology, The First Affiliated Hospital of Xinxiang Medical University, Xinxiang, China; 16 Department of Thoracic Surgery, Yijishan Hospital of Wannan Medical College, Wuhu, China; 17 Department of Gastroenterology, Harbin Medical University Cancer Hospital, Harbin, China; 18 Department of Gastroenterology, Ganzhou People’s Hospital - North Hospital, Ganzhou, China; 19 Department of Medial Oncology, Nanyang Central Hospital, Nanyang, China; 20 Department of Medical Affairs, Jiangsu Hengrui Pharmaceuticals Co., Ltd., Shanghai, China

**Keywords:** Esophageal cancer, Immunotherapy, Real-world, Overall survival

## Abstract

**Background and aims:**

This individual patient data pooled analysis aimed to evaluate the effectiveness, safety, and patterns of use of camrelizumab in a large cohort of advanced esophageal cancer (AEC) patients.

**Approach and results:**

Adult patients (≥ 18 years) who had received camrelizumab as part of AEC treatment were pooled from three independent, prospective observational cohort studies (NCT04616040, ChiCTR1900027275, and ChiCTR2000039499). The main outcomes were patterns of camrelizumab use, progression-free survival (PFS), overall survival (OS), and safety in the overall population and specific subgroups of underrepresented patients. Among 987 patients, 450 (45.6%) received camrelizumab in the first line, 398 (40.3%) in the second line, and 139 (14.1%) in the third line or later. Most (69.7%) patients received camrelizumab plus chemotherapy regardless of treatment lines. The median PFS was 9.9 (95% CI 7.4, 14.4), 6.6 (95% CI 5.1, 8.8), and 5.7 (95% CI 3.1, 9.6) months in the first line, second line, and third line or later, respectively. The corresponding median OS was 15.5 (95% CI 12.6, 18.4), 12.1 (95% CI 10.0, 14.7), and 10.9 (95% CI 8.1, 14.5) months. Patients with poor performance status (ECOG PS ≥ 2) and with camrelizumab in the second line or later, but not patients with older age (≥ 75 years), were associated with poor survival. Adverse events occurred in 721 (73.0%) patients, with no new safety signals.

**Conclusions:**

This study provides an overview of camrelizumab use in unselected AEC patients. The real-world effectiveness and safety of camrelizumab are generally consistent with those observed in pivotal trials.

**Supplementary Information:**

The online version contains supplementary material available at 10.1007/s00262-025-03970-z.

## Introduction

Esophageal cancer is one of the leading causes of cancer-related deaths worldwide, with approximately 511,000 new cases and 445,000 deaths in 2022 [1]. More than half of the new cases and deaths are estimated to occur in China [2]. Most patients diagnosed with esophageal cancer are at an advanced stage. The estimated 5-year survival rate is around 10–30% [3]. Immunotherapy with immune checkpoint inhibitors (ICIs) has changed the treatment landscape of patients with advanced esophageal cancer. Several programmed cell death protein 1 (PD-1) or its ligand (PD-L1) inhibitors, such as pembrolizumab and nivolumab, have been approved for clinical use [4–8].

Camrelizumab (SHR-1210), a humanized PD-1 monoclonal antibody, has been approved either alone or in combination by the National Drug Administration (NMPA) in China for the treatment of advanced or metastatic esophageal squamous cell cancer (ESCC). The phase III ESCORT-1st trial confirmed the efficacy and safety of camrelizumab in combination with paclitaxel and cisplatin chemotherapy for the first-line treatment of advanced or metastatic ESCC [9]. In addition, camrelizumab in combination with apatinib (a selective vascular endothelial growth factor receptor-2 [VEGFR-2] inhibitor) and chemotherapy (liposomal paclitaxel and nedaplatin) exhibited anti-tumor activity as the first-line treatment in patients with advanced ESCC [10]. In the second-line setting, the phase III ESCORT trial demonstrated improved survival with camrelizumab monotherapy versus investigator-choice chemotherapy in patients with advanced or metastatic ESCC who had progressed on or were intolerant to prior first-line chemotherapy [11]. Moreover, camrelizumab in combination with apatinib showed promising activity and manageable safety as the second-line therapy in patients with advanced, recurrent or metastatic ESCC [12]. Based on these results, camrelizumab in combination with chemotherapy with or without apatinib has been recommended for the first-line treatment of advanced or metastatic esophageal cancer, while camrelizumab with or without apatinib has been recommended for the second-line treatment of advanced or metastatic esophageal cancer after progressed on prior first-line chemotherapy in the Chinese Society of Clinical Oncology (CSCO) guidelines.

However, the real-world effectiveness and safety of camrelizumab in patients with advanced esophageal cancer treated in daily practice have not been extensively investigated. The available evidence is primarily based on patients meeting strict screening criteria and receiving predefined treatment regimens in pivotal clinical trials. It may not fully reflect real-world patient care practices and outcomes. Three prospective multicenter observational cohort studies have investigated the real-world effectiveness and safety of camrelizumab in patients with advanced esophageal cancer [13–15]. This study was therefore designed to pool individual patient data (IPD) from the above-mentioned three observational studies to further evaluate the real-world effectiveness, safety, and patterns of use of camrelizumab in a large sample of patients with advanced esophageal cancer. The IPD pooled analysis may not only provide more precision estimation but also enrich specific patients that are underrepresented in pivotal clinical trials (e.g., patients aged ≥ 75 years or with Eastern Cooperative Oncology Group [ECOG] performance status of ≥ 2). The findings of this study may help to better understand the current treatment practice and outcomes in advanced esophageal cancer patients across different treatment lines and those with specific clinical characteristics.

## Methods

### Study design and patients

This was a pooled analysis of IPD from three independent, large-scale, prospective multicenter observational cohort studies in China (Trial 1: ESCORT-RWS/NCT04616040, Trial 2: NOAH-EC201/ChiCTR1900027275, and Trial 3: ChiCTR2000039499). The studies were similar in study design enabling post hoc pooled analysis (Supplementary Table 1). All three studies were designed to evaluate the real-world effectiveness and safety of camrelizumab for the treatment of patients with unresectable locally advanced, recurrent, or metastatic esophageal cancer. All consecutive patients who met the eligibility criteria were screened for potential participation in the individual studies to minimize selection bias. Patients included were 18 years or older and had received at least one dose of camrelizumab as part of advanced esophageal cancer treatment. All patients were managed and followed at the discretion of the physician-in-charge according to routine clinical practice and patient preference.

All three studies had been approved by the institutional review boards (No. 2020YJZ57-GZ03; No. PJ2019-12-07; and No. ChiECRCT20190176).

### Procedures

This study included all patients from the above three cohort studies. IPD were obtained from December 24, 2020, to December 30, 2022, for trial one, from July 30, 2019, to November 30, 2020, for trial two, and from October 1, 2019 to January 7, 2022, for trial three. The data cut-off date for the study was December 30, 2022. IPD were recorded in an electronic data capture (EDC) system for trial one. Data from trials two and three were retrieved and imported into the EDC based on the Clinical Data Interchange Standards Consortium (CDISC) standards. De-identified data were pooled for baseline demographic (sex, age, body mass index, smoking, and drinking status) and clinical characteristics (Eastern Cooperative Oncology Group [ECOG] performance status, histological type, primary tumor location, and prior antitumor therapy), peripheral blood parameters, camrelizumab treatment characteristics (patterns of use, treatment cycles, time and reasons for treatment discontinuation), and effectiveness and safety outcomes. Adverse events were collected from the date of camrelizumab initiation to the date of the end of the study. Since this study was conducted during the COVID-19 pandemic, data on COVID-19 infection were also collected.

### Outcomes

The primary objective was to evaluate the pooled effectiveness and safety of camrelizumab in advanced esophageal cancer patients. The study outcomes included patterns of camrelizumab use, progression-free survival (PFS, the time from the start of camrelizumab to the investigator-assessed disease progression or death from any cause, whichever occurred first), overall survival (OS, the time the start of camrelizumab to death from any cause), time to treatment discontinuation (TTD, the time from the start of camrelizumab to treatment discontinuation for any reason), objective response rate (ORR, the percentage of patients with investigator-assessed complete response [CR] or partial response [PR]), disease control rate (DCR, the percentage of patients with investigator-assessed CR, PR, or stable disease), and adverse events. In addition, other objectives were pooled effectiveness and safety in specific subgroups of patients who were underrepresented in the pivotal clinical trials (age ≥ 75 years or an ECOG performance status ≥ 2), as well as pooled effectiveness with different patterns of camrelizumab use (different systemic therapy: camrelizumab alone or in combination with chemotherapy, antiangiogenic therapy, or chemotherapy and antiangiogenic therapy; different chemotherapy: taxanes plus platinum [TP], fluorouracil plus platinum [FP], and others; or different paclitaxel-based chemotherapy: paclitaxel, nanoparticle albumin-bound [nab]-paclitaxel, and docetaxel).

### Statistical analysis

Effectiveness analyses were primarily performed in the full analysis set (FAS, all patients who had received at least one dose of camrelizumab). Tumor response was analyzed in the effectiveness analysis set (EAS, patients from the FAS population who had at least one post-baseline tumor assessment). Safety analyses were performed in the safety analysis set (SAS, all patients from the FAS population).

Continuous variables were expressed as mean ± standard deviation (SD) or median and range or interquartile range (IQR). Categorical variables were expressed as frequency and percentage. Time-to-event outcomes (PFS, OS, and TTD) were calculated using the Kaplan–Meier method, and corresponding 95% confidence intervals (CIs) were estimated using the Brookmeyer-Crowley method. The binary outcomes (ORR and DCR) were expressed as frequency and percentage, and corresponding 95% CIs were estimated using the Clopper-Pearson method. Multiple multivariate Cox regression analyses were performed to evaluate the impact of peripheral blood parameters on OS by adjusting for baseline patient characteristics (age, sex, smoking status, drinking status, primary tumor location, liver metastases, lung metastases, bone metastases, line of therapy, and camrelizumab treatment patterns). Peripheral blood parameters were classified into two groups (normal and abnormal) according to their previously established normal value or range in the Cox regression analyses. A correlation test between the weighted residuals and failure times was used for checking the proportional hazards assumption. If the proportional hazards assumption was not met in the raw Cox model, the time-dependent covariate CovT = Cov*(log(time)) would be introduced in the final Cox model. The hazard ratio (HR) with profile-likelihood confidence limits was calculated to estimate the predictive values of the parameters stratified by normal versus abnormal baseline values. The Benjamini and Hochberg method was used to control the false discovery rate (FDR) in multiple comparisons. All analyses were performed using SAS 9.4 (SAS Institute Inc.).

## Results

### Patients and site characteristics

**Fig. 1:**
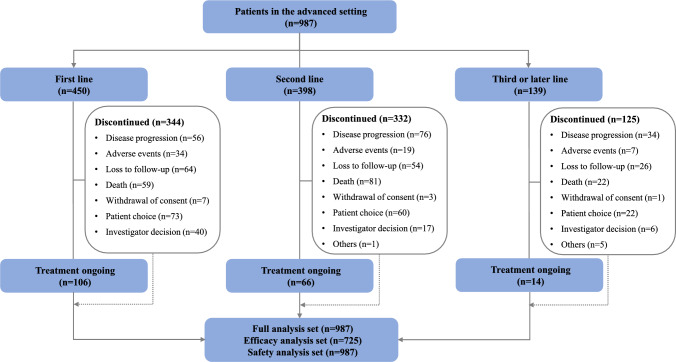
Patient flowchart

This pooled analysis included 987 patients (624 from trial one; 229 from trial two; 134 from trial three) from 91 centers across 20 provinces in China (Supplementary Figure 1). Most centers were general hospitals (84.6%) and tertiary care hospitals (86.8%) (Supplementary Table 2). There was no overlap in patients among the three studies. All 987 patients were included in the FAS and SAS analyses, and 725 (73.5%) patients who underwent at least one post-baseline tumor assessment were included in the EAS analysis (Fig. 1).

The median age of the pooled population was 65 (range: 26, 92) years, and 13.3% (131/987) were aged ≥ 75 years. Most patients were male (81.9%), had an ECOG performance status of 0 or 1, had squamous cell carcinoma (96.0%), and had tumors located in the thoracic esophagus (91.6%). Sixty-three (6.4%) patients had an ECOG performance status of ≥ 2. Thirty-six (3.6%) patients had previously received ICIs (31 [86.1%] anti-PD-1 inhibitors).

Of the 987 patients, 450 (45.6%) received camrelizumab in the first line, 398 (40.3%) in the second line, and 139 (14.1%) in the third line or later. The baseline characteristics of the patients with the different lines of camrelizumab treatment are shown in Table 1.

**Table 1:** Baseline patient characteristics (n = 987)

	1st line (n = 450)	2nd line (n = 398)	3rd line or later (n = 139)
Median age (range), years	66 (26–92)	64 (30–86)	64 (45–79)
≥ 75 years, n (%)	67 (14.9)	49 (12.3)	15 (10.8)
Sex, n (%)			
Male	366 (81.3)	328 (82.4)	114 (82.0)
Female	84 (18.7)	70 (17.6)	25 (18.0)
Mean BMI (± SD), kg/m^2^	21.3 (3.4)	20.8 (3.1)	20.8 (2.9)
Smoking, n (%)			
Never	211 (46.9)	209 (52.5)	67 (48.2)
Former	124 (27.6)	107 (26.9)	41 (29.5)
Current	94 (20.9)	51 (12.8)	20 (14.4)
Unknown	21 (4.7)	31 (7.8)	11 (7.9)
Alcohol consumption, n (%)			
Never	245 (54.4)	224 (56.3)	74 (53.2)
Former	97 (21.6)	86 (21.6)	35 (25.2)
Current	82 (18.2)	53 (13.3)	17 (12.2)
Unknown	25 (5.8)	35 (8.8)	13 (9.4)
ECOG PS, n (%)			
0–1	385 (89.5)	340 (89.2)	108 (81.2)
≥ 2	21 (4.9)	24 (6.3)	18 (13.5)
Unknown	24 (5.6)	17 (4.5)	7 (5.3)
Histology, n (%)			
Squamous cell carcinoma	430 (95.6)	383 (96.2)	135 (97.1)
Adenocarcinoma	4 (0.9)	3 (0.8)	2 (1.4)
Others	14 (3.1)	7 (1.8)	1 (0.7)
Unknown	2 (0.4)	5 (1.3)	1 (0.7)
Primary tumor location, n (%)			
Cervical esophagus	14 (3.1)	9 (2.3)	2 (1.4)
Upper thoracic esophagus	83 (18.4)	65 (16.3)	22 (15.8)
Middle thoracic esophagus	223 (49.6)	222 (55.8)	75 (54.0)
Lower thoracic esophagus	107 (23.8)	80 (20.1)	27 (19.4)
Others	4 (0.9)	2 (0.5)	7 (5.0)
Unknown	19 (4.2)	20 (5.0)	6 (4.3)
Prior ICIs, n (%)	4 (0.4)	10 (1.0)	22 (2.2)

*BMI* Body mass index; *SD* standard deviation, *ECOG PS* Eastern Cooperative Oncology Group performance status, *ICIs* Immune checkpoint inhibitors

### Camrelizumab treatment characteristics

As of December 30, 2022, the median number of cycles of camrelizumab therapy was 4 (range 1–42), 5 (range 1–52), and 3 (range 1–43) in the first line, second line, and third line or later, respectively. Camrelizumab combined with chemotherapy (69.7%) was most frequently prescribed regardless of treatment lines, primarily TP-based chemotherapy, followed by camrelizumab combined with antiangiogenic therapy (12.5%), camrelizumab monotherapy (11.2%), and camrelizumab combined with chemotherapy and antiangiogenic therapy (6.6%) (Table 2). A total of 141 (14.3%) patients received concomitant radiotherapy. Ninety-six (9.7%) patients continued camrelizumab treatment beyond progression.

**Table 2:** Camrelizumab treatment in the full analysis set (n = 987)

	1st line (n = 450)	2nd line (n = 398)	3rd line or later (n = 139)
Camrelizumab treatment, cycles			
Median (range)	4 (1, 42)	5 (1, 52)	3 (1, 43)
Patterns of camrelizumab use, n (%)			
Monotherapy	31 (6.9)	63 (15.8)	17 (12.2)
Plus chemotherapy	379 (84.2)	239 (60.3)	70 (50.4)
Taxane and platinum regimens	304 (80.2)	142 (59.2)	35 (50.0)
Fluoropyrimidine and platinum regimens	63 (16.6)	72 (30.0)	24 (34.3)
Others	12 (3.2)	25 (10.4)	11 (15.7)
Plus antiangiogenic therapy	19 (4.2)	63 (15.8)	41 (29.5)
Plus chemotherapy and antiangiogenic therapy	21 (4.7)	33 (8.3)	11 (7.9)
Antiangiogenic agents use, n (%)*			
Tyrosine kinase inhibitors	34 (85.0)	89 (92.7)	50 (96.2)
Apatinib	25 (62.5)	49 (51.0)	32 (61.5)
Anlotinib	9 (22.5)	40 (41.7)	18 (34.6)
Others	6 (15.0)	7 (7.3)	2 (3.8)
Concomitant radiotherapy	86 (19.1)	46 (11.6)	9 (6.5)
Camrelizumab treatment beyond progression, n (%)	0	33 (8.3)	63 (45.3)

“*” indicates among patients receiving antiangiogenic therapy

At the data cutoff, 801 (81.2%) of the 987 patients permanently discontinued camrelizumab treatment. The main reasons for treatment discontinuation were disease progression (20.7%), death (20.2%), and patient choices (19.4%).

### Effectiveness in the overall population

**Fig. 2:**
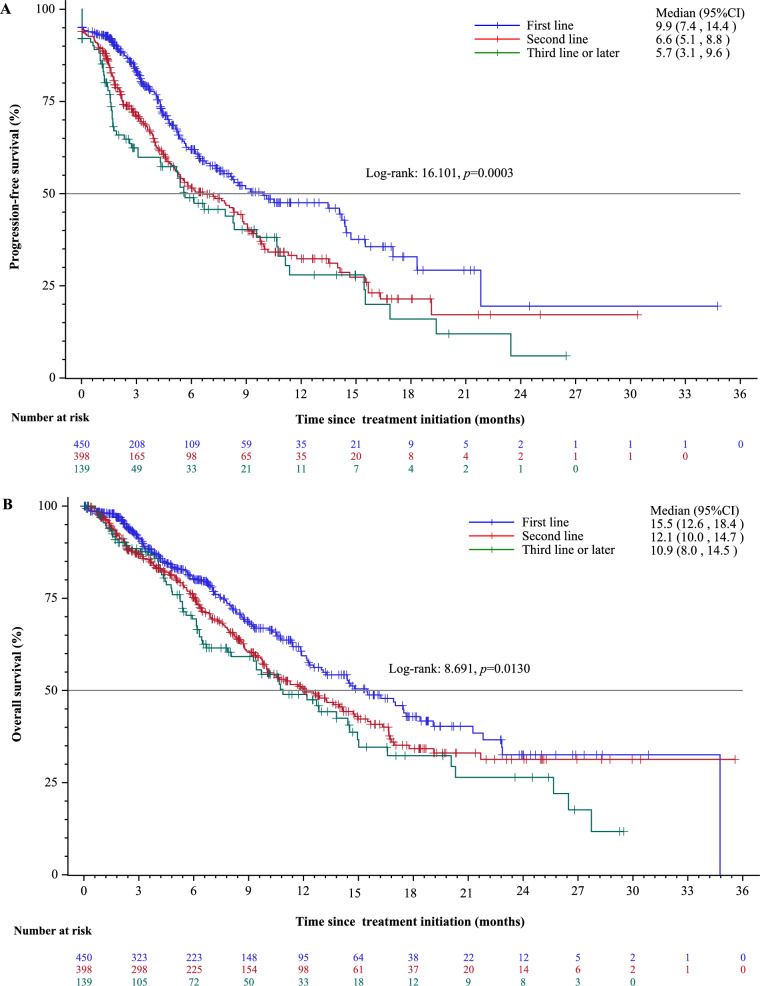
Kaplan–Meier curves for progression-free survival (**A**) and overall survival (**B**)

The median follow-up was 8.7 (95% CI 7.1, 9.6) months in the first line, 12.0 (95% CI 10.4, 12.9) months in the second line, and 11.7 (95% CI 9.1, 13.8) months in the third line or later. A total of 359 (36.4%) patients experienced disease progression or had died without disease progression, including 129 (28.7%) in the first line, 166 (41.7%) in the second line, and 64 (46.0%) in the third line or later. The median PFS was 9.9 (95% CI 7.4, 14.4) in the first line, 6.6 (95% CI 5.1, 8.8) in the second line, and 5.7 (95% CI 3.1, 9.6) months in the third line or later (Fig. 2A).

During the follow-up, 385 patients had died, including 142 (31.6%) in the first line, 175 (44.0%) in the second line, and 68 (48.9%) in the third line or later. The median OS was 15.5 (95% CI 12.6, 18.4), 12.1 (95% CI 10.0, 14.7), and 10.9 (95% CI 8.1, 14.5) months, respectively (Fig. 2B). Among the 801 patients who discontinued camrelizumab treatment, 344 were in the first line, 332 in the second line, and 125 in the third line or later. The corresponding median TTD was 4.8 (95% CI 4.1, 5.6), 4.8 (95% CI 3.8, 5.4), and 2.9 (95% CI 2.2, 4.3) months, respectively (Supplementary Figure 2).

In the EAS analysis of 725 patients, the ORR was 45.5% (95% CI 40.0, 51.0) in the first line, 29.0% (95% CI 23.9, 34.5) in the second line, and 22.9% (95% CI 15.0, 32.6) in the third line or later, as shown in Table 3. The corresponding DCR was 90.1% (95% CI 86.3, 93.1), 79.5% (95% CI 74.4, 83.9), and 68.8% (95% CI 58.5, 77.8), respectively.

**Table 3:** Tumor response in efficacy analysis set (n = 725)

	Efficacy analysis set		
	1st line (n = 332)	2nd line (n = 297)	3rd line or later (n = 96)
Objective response, n (%)	151 (45.5)	86 (29.0)	22 (22.9)
95% CI	40.0, 51.0	23.9, 34.5	15.0, 32.6
Disease control, n (%)	299 (90.1)	236 (79.5)	66 (68.8)
95% CI	86.3, 93.1	74.4, 83.9	58.5, 77.8
Best overall response, n (%)			
Complete response	9 (2.7)	6 (2.0)	2 (2.1)
Partial response	142 (42.8)	80 (26.9)	20 (20.8)
Stable disease	148 (44.6)	150 (50.5)	44 (45.8)
Progressive disease	23 (6.9)	58 (19.5)	29 (30.2)
Not evaluable	10 (3.0)	3 (1.0)	1 (1.0)

### Effectiveness in older patients and those with poor performance status

The results of subgroup analyses for PFS and OS are summarized in Supplementaty Figures 3–6. Patients aged ≥ 75 years were not associated with poor PFS or OS when compared with the overall population and those younger (≥ 75 vs 65 ~ 75 vs < 65 years, median PFS: 14.4 [95% CI 5.3, NR] vs 13.5 [95% CI 7.4, 15.5] vs 8.1 [95% CI 5.7, 17.0] months; median OS: 14.4 [95% CI 6.9, NR] vs 19.1 [95% CI 12.2, 22.9] vs 14.8 [95% CI 11.9, 17.5] months) in the first line setting (Supplementary Figures 7A and 7B). A similar trend was observed in the second line or later setting (Supplementary Figures 8A and 8B).

In the first-line setting, patients with an ECOG performance status of ≥ 2 were not associated with poor PFS (median: 10.5 [95% CI 3.2, 14.2] vs 9.2 [95% CI 6.8, 14.7] months) or OS (median: 21.8 [95% CI 4.1, NR] vs 14.8 [95% CI 12.3, 17.6] months) when compared with patients with a performance status of 0 or 1 (Supplementary Figures 9A and 9B). By contrast, in the second line or later setting, patients with a performance status of ≥ 2 showed shorter median PFS (3.1 [95% CI 1.6, 5.4] vs 6.6 [95% CI 5.3, 8.8] months) and OS (4.1 [95% CI 3.1, 8.8] vs 12.7 [95% CI 10.6, 14.7] months) (Supplementary Figure 10A and 10B).

### Effectiveness with different camrelizumab treatment regimens

In the first-line setting, camrelizumab combined with chemotherapy was associated with impressive PFS (median: 13.5 [95% CI 7.8, 17.0] vs 8.2 [95% CI 2.0, NR] vs 4.8 [95% CI 2.6, NR] months) and OS (median: 17.4 [95% CI 13.1, 21.8] vs 8.5 [95% CI 3.2, 17.0] vs 10.5 [95% CI 4.8, NR] months) when compared with camrelizumab combined with anti-angiogenic therapy with or without chemotherapy (Supplementary Figures 3 and 4). As for the different chemotherapy regimens, camrelizumab combined with the TP regimen resulted in numerically longer PFS (median: 14.4 [95% CI 7.8, 18.3] vs 8.2 [95% CI 4.7, 14.2] months) and OS (median: 17.5 [95% CI 13.1, 21.3] vs 14.8 [95% CI 7.8, NR] months) when compared with camrelizumab combined with the FP regimen (Supplementary Figures 11A and 11B). When came to the different TP regimens, the PFS and OS were generally similar.

In the second line or later setting, camrelizumab monotherapy showed numerically prolonged median survival when compared with camrelizumab combined with chemotherapy or antiangiogenic therapy (Supplementary Figures 5 and 6). However, no significant differences were observed between the groups (nominal Log-rank *p* = 0.5091 and *p* = 0.2086 for PFS and OS, respectively) (Supplementary Figures 12).

### Safety

Among the 987 patients in the SAS population, 721 (73.0%) patients experienced at least one adverse event. The most common adverse events were anemia (29.9%), decreased white blood cell count (27.9%), and decreased neutrophil count (19.6%) (Supplementary Table 3). Sixty-four (5.2%) patients developed COVID-19 infection. There were no new safety signals. Similar safety profiles were observed in patients aged ≥ 75 years (Supplementary Table 4) and those with an ECOG performance status of ≥ 2 (Supplementary Table 5).

### Peripheral blood biomarkers

Multiple multivariate Cox regression analyses were performed to identify potential peripheral blood biomarkers that may be associated with OS of patients (Supplementary Table 6). After adjusting for baseline patient characteristics and multiple comparisons, lymphocyte-to-monocyte ratio (LMR) (HR: 0.22, 95% CI 0.09, 0.50, FDR-adjusted *p* = 0.011), albumin (ALB) (HR: 0.54, 95% CI 38, 0.78, FDR-adjusted *p* = 0.011), platelet-to-lymphocyte ratio (PLR) (HR: 0.36, 95% CI 0.18, 0.67, FDR-adjusted *p* = 0.023), and neutrophil-to-lymphocyte ratio (NLR) (HR: 0.65, 95% CI 0.48, 0.88, FDR-adjusted *p* = 0.039) remained significantly associated with OS (Supplementary Table 7).

## Discussion

With the approvals of PD-1/PD-L1 inhibitors in esophageal cancer, clinicians are now faced with substantial new questions concerning effectiveness, safety, and optimal treatment patterns in a broader patient population we deal with in daily life, including fragile patients with older age and those with poor performance status that are generally excluded from pivotal trials. This study provides valuable insights into the effectiveness, safety, and patterns of use of camrelizumab in advanced esophageal cancer outside of a clinical trial, on the basis of an IPD pooled population of almost 1000 patients from 91 centers across 20 provinces in China. Camrelizumab combined with chemotherapy was most commonly prescribed irrespective of the line of therapy, which is a little bit going beyond what would be expected from guideline-recommended patient care. Camrelizumab monotherapy or in combination with apatinib has been recommended as the second-line treatment for patients with advanced esophageal cancer in China. The median OS was 15.5, 12.1, and 10.9 months in our unselected patients receiving camrelizumab in the first, second, and third or later lines, respectively. The safety profile was aligned with the known profiles of camrelizumab alone or in combination with other agents, with no new safety signals noted. The findings of our study support the effectiveness and safety of camrelizumab in real-world patients with advanced esophageal cancer.

The real-world effectiveness of camrelizumab in our advanced esophageal cancer patients is generally consistent with that observed in pivotal clinical trials of camrelizumab [9–12] (Supplementary Table 8) and that of nivolizumab in the previously heavily treated patients with advanced ESCC [16], while was better than that of pembrolizumab in heavily pretreated adenocarcinoma or squamous cell carcinoma of the esophagus [17], although the results from observational studies and clinical trials are not directly comparable [18, 19]. Unlike the predefined populations in pivotal trials, a subset of fragile patients aged ≥ 75 years (13.3%) and those with an ECOG performance status of ≥ 2 (6.7%) who would not have met the trial eligibility criteria were included in this study. Unlike the highly protocolized patient care in pivotal trials, the usual patient care is imbued with varying treatment patterns and no consistent time points and criteria for radiological response assessment in real-world settings, which may also have affected the observed outcomes, especially ORR and PFS. Nevertheless, the OS, a robust endpoint, observed in our study was generally consistent with that of the pivotal trials, supporting the effectiveness of camrelizumab in real-world patients.

Importantly, clinical outcomes in patients aged ≥ 75 years were similar to those in the overall population and younger patients. However, patients with a performance status of ≥ 2 and with camrelizumab in the second line or later, but not in the first line, had shorter PFS and OS when compared with their counterparts with a performance status of 0 or 1, regardless of similar safety profiles. These findings are novel, as data on the effectiveness of immunotherapy in these specific subgroups of patients have been lacking. Few very small retrospective studies reported no significant impact of poor performance status on tumor response and survival in advanced ESCC treated with ICIs [20, 21]. Additionally, in a previous study of first-line pembrolizumab in advanced non-small cell lung cancer, outcomes of patients with a performance status of 2 were strongly dependent on the reasons conditioning the poor performance status itself [22]. However, the number of patients with poor performance status was still limited. Future studies with a larger sample size are, therefore, warranted to validate the findings observed here and to explore the reasons behind the poor performance status and their influences on the prognosis of patients during immunotherapy.

Both TP and FP regimens have been first-line backbone chemotherapy for chemoimmunotherapy combinations in advanced esophageal cancer. However, there are regional differences in the choice of chemotherapy regimen. The TP regimen is more common in Asia, particularly in China, while the FP regimen is preferred in the West and Japan. Efficacy data suggest a trend toward better survival with TP regimens over FP regimens [23, 24]. Currently, ICIs in combination with TP or FP (TP: camrelizumab [9], Toripalimab [25]; FP: pembrolizumab [4], nivolumab [5, 16]; TP/FP: sintilimab [26], Tislelizumab [27]) have been recommended for the first-line treatment of advanced esophageal cancer in China. TP regimen appears to derive more survival benefit over the FP regimen when in combination with immunotherapy [28]. In our study, 63 (14.0%) out of 450 patients received first-line FP combined with camrelizumab. The median OS was numerically better with camrelizumab combined with TP regimens than that of camrelizumab combined with FP regimens, corroborating the findings of a previous meta-analysis [29]. However, we could not account for the potential differences in baseline patient characteristics, which may have some effects on survival outcomes. Nevertheless, the median OS of 14.8 months with camrelizumab combined with FP regimens observed in this study is superior to historical data of 9.8–11.6 months with FP chemotherapy alone in the KEYNOTE-590, CheckMate 648, and CheckMate 649 trials [4, 5, 16], supporting the camrelizumab combined with FP chemotherapy as an alternative option for the first line treatment of advanced esophageal cancer.

We documented adverse events in 73.0% of our patients. The safety profile was in line with the known profile of camrelizumab or in combination with chemotherapy and/or antiangiogenic therapy, with no new safety signals observed [9–12]. However, the incidence of adverse events, such as RCCEP, was fairly low in our real-world setting when compared with those in the pivotal trials [9, 11], although the data were generally consistent with previous research on camrelizumab in the other real-world settings (e.g., NSCLC and esophageal cancer, RCCEP: 6.9–39.2%) [30–32]. Additionally, data on the severity of adverse events were not routinely documented. Observational studies may be biased by underestimation of adverse events, especially those mild in intensity.

We also explored the associations between peripheral blood parameters and survival in patients with advanced esophageal cancer treated with camrelizumab. After adjusting for baseline patient characteristics and multiple comparisons in multiple multivariate Cox regression analyses, baseline normal LMR, albumin, PLR, and NLR were significantly associated with better OS. These results are generally consistent with our previous studies [33, 34] and those of other investigators [35–38], suggesting that these easy-access biomarkers might be useful for prognostic prediction in patients with advanced esophageal cancer treated with ICIs.

This study also has some limitations, primarily, the observational nature of the study design that may introduce potential bias, although it is better able to capture the real-world data. The cross-study comparisons should be interpreted with caution. Besides, we mainly focus on camrelizumab in China, patients were mainly from tertiary or secondary hospitals in the southeast areas, so the results observed may not be generalized to all patients and the wider class of PD-1/PD-L1 inhibitors used in clinical practice. We mainly focused on the effectiveness and patterns of camrelizumab use in this study, only the treatment emergent adverse events were reported regarding safety. The detailed safety data including immune-related adverse events would be reported in our future work. In addition, potential biomarkers such as PD-L1 expression were not routinely determined in daily practice and therefore not included as an adjustment factor in the multivariate analysis. Given the predictive value of PD-L1 expression in immunotherapy in advanced esophageal cancer, its omission could potentially confound the results.

Taken together, this study based on a large sample size provides an overview of real-world camrelizumab use in advanced esophageal cancer patients. The real-world effectiveness of camrelizumab in unselected patients with advanced esophageal cancer is generally consistent with that observed in pivotal trials, with no new safety signal noted. Patients aged ≥ 75 years had similar survival outcomes to the overall population and those younger. Patients with a performance status of ≥ 2 and with camrelizumab in the second line or later had poor survival. In addition to TP chemotherapy, camrelizumab combined with FP chemotherapy may be an alternative option for the first-line treatment of advanced esophageal cancer in the real-world setting.

## Supplementary Information

Below is the link to the electronic supplementary material.

Supplementary file1

## Data Availability

The data that support the findings of this study are available on request from the corresponding authors.

## References

[1] Bray F, Laversanne M, Sung H, Ferlay J, Siegel RL, Soerjomataram I, Jemal A (2024) Global cancer statistics 2022: GLOBOCAN estimates of incidence and mortality worldwide for 36 cancers in 185 countries. CA Cancer J Clin. 10.3322/caac.21834 38572751 10.3322/caac.21834

[2] Morgan E, Soerjomataram I, Rumgay H, Coleman HG, Thrift AP, Vignat J, Laversanne M, Ferlay J, Arnold M (2022) The global landscape of esophageal squamous cell carcinoma and esophageal adenocarcinoma incidence and mortality in 2020 and projections to 2040: new estimates from GLOBOCAN 2020. Gastroenterology 163:649–58.e2. 10.1053/j.gastro.2022.05.054 35671803 10.1053/j.gastro.2022.05.054

[3] Allemani C, Matsuda T, Di Carlo V et al (2018) Global surveillance of trends in cancer survival 2000–14 (CONCORD-3): analysis of individual records for 37 513 025 patients diagnosed with one of 18 cancers from 322 population-based registries in 71 countries. Lancet 391:1023–1075. 10.1016/s0140-6736(17)33326-3 29395269 10.1016/S0140-6736(17)33326-3 PMC5879496

[4] Sun JM, Shen L, Shah MA et al (2021) Pembrolizumab plus chemotherapy versus chemotherapy alone for first-line treatment of advanced oesophageal cancer (KEYNOTE-590): a randomised, placebo-controlled, phase 3 study. Lancet 398:759–771. 10.1016/s0140-6736(21)01234-4 34454674 10.1016/S0140-6736(21)01234-4

[5] Doki Y, Ajani JA, Kato K et al (2022) Nivolumab combination therapy in advanced esophageal squamous-cell carcinoma. N Engl J Med 386:449–462. 10.1056/NEJMoa2111380 35108470 10.1056/NEJMoa2111380

[6] Janjigian YY, Shitara K, Moehler M et al (2021) First-line nivolumab plus chemotherapy versus chemotherapy alone for advanced gastric, gastro-oesophageal junction, and oesophageal adenocarcinoma (CheckMate 649): a randomised, open-label, phase 3 trial. Lancet 398:27–40. 10.1016/s0140-6736(21)00797-2 34102137 10.1016/S0140-6736(21)00797-2 PMC8436782

[7] Kato K, Cho BC, Takahashi M et al (2019) Nivolumab versus chemotherapy in patients with advanced oesophageal squamous cell carcinoma refractory or intolerant to previous chemotherapy (ATTRACTION-3): a multicentre, randomised, open-label, phase 3 trial. Lancet Oncol 20:1506–1517. 10.1016/s1470-2045(19)30626-6 31582355 10.1016/S1470-2045(19)30626-6

[8] Kojima T, Shah MA, Muro K et al (2020) Randomized phase III KEYNOTE-181 study of pembrolizumab versus chemotherapy in advanced esophageal cancer. J Clin Oncol 38:4138–4148. 10.1200/jco.20.01888 33026938 10.1200/JCO.20.01888

[9] Luo H, Lu J, Bai Y et al (2021) Effect of camrelizumab vs placebo added to chemotherapy on survival and progression-free survival in patients with advanced or metastatic esophageal squamous cell carcinoma: the ESCORT-1st randomized clinical trial. JAMA 326:916–925. 10.1001/jama.2021.12836 34519801 10.1001/jama.2021.12836 PMC8441593

[10] Zhang B, Qi L, Wang X, Xu J, Liu Y, Mu L, Wang X, Bai L, Huang J (2020) Phase II clinical trial using camrelizumab combined with apatinib and chemotherapy as the first-line treatment of advanced esophageal squamous cell carcinoma. Cancer Commun (Lond) 40:711–720. 10.1002/cac2.12119 33314747 10.1002/cac2.12119 PMC7743020

[11] Huang J, Xu J, Chen Y et al (2020) Camrelizumab versus investigator’s choice of chemotherapy as second-line therapy for advanced or metastatic oesophageal squamous cell carcinoma (ESCORT): a multicentre, randomised, open-label, phase 3 study. Lancet Oncol 21:832–842. 10.1016/s1470-2045(20)30110-8 32416073 10.1016/S1470-2045(20)30110-8

[12] Meng X, Wu T, Hong Y et al (2022) Camrelizumab plus apatinib as second-line treatment for advanced oesophageal squamous cell carcinoma (CAP 02): a single-arm, open-label, phase 2 trial. Lancet Gastroenterol Hepatol 7:245–253. 10.1016/s2468-1253(21)00378-2 34998471 10.1016/S2468-1253(21)00378-2

[13] Lu Z, Zhao J, Yang Z et al (2023) Effectiveness and safety of camrelizumab in advanced esophageal cancer: a prospective multicenter observational cohort studies (ESCORT-RWS). J Clin Oncol 41:4049. 10.1200/JCO.2023.41.16_suppl.4049

[14] Yao Q, Fu Z, Chen Q et al (2021) Real-world clinical effectiveness and safety of camrelizumab in esophageal squamous cell carcinoma. J Clin Oncol 39:e16023. 10.1200/JCO.2021.39.15_suppl.e16023

[15] Sun G, Qian D, Zhao D et al (2021) A real-world clinical study of camrelizumab in the treatment of esophageal cancer. J Clin Oncol 39:e16008. 10.1200/JCO.2021.39.15_suppl.e16008

[16] Kudo T, Hamamoto Y, Kato K et al (2017) Nivolumab treatment for oesophageal squamous-cell carcinoma: an open-label, multicentre, phase 2 trial. Lancet Oncol 18:631–639. 10.1016/s1470-2045(17)30181-x 28314688 10.1016/S1470-2045(17)30181-X

[17] Shah MA, Kojima T, Hochhauser D et al (2019) Efficacy and safety of pembrolizumab for heavily pretreated patients with advanced, metastatic adenocarcinoma or squamous cell carcinoma of the esophagus: the phase 2 KEYNOTE-180 study. JAMA Oncol 5:546–550. 10.1001/jamaoncol.2018.5441 30570649 10.1001/jamaoncol.2018.5441 PMC6459121

[18] Wang SV, Schneeweiss S, Franklin JM et al (2023) Emulation of randomized clinical trials with nonrandomized database analyses: results of 32 clinical trials. JAMA 329:1376–1385. 10.1001/jama.2023.4221 37097356 10.1001/jama.2023.4221 PMC10130954

[19] Franklin JM, Patorno E, Desai RJ et al (2021) Emulating randomized clinical trials with nonrandomized real-world evidence studies: first results from the RCT DUPLICATE initiative. Circulation 143:1002–1013. 10.1161/circulationaha.120.051718 33327727 10.1161/CIRCULATIONAHA.120.051718 PMC7940583

[20] Kao MW, Kuo YH, Hsieh KC, Lee CT, Wu SC, Yang WC (2023) Immune checkpoint inhibitor, nivolumab, combined with chemotherapy improved the survival of unresectable advanced and metastatic esophageal squamous cell carcinoma: a real-world experience. Int J Mol Sci. 10.3390/ijms24087312 37108474 10.3390/ijms24087312 PMC10139434

[21] Kim JH, Ahn B, Hong SM et al (2022) Real-world efficacy data and predictive clinical parameters for treatment outcomes in advanced esophageal squamous cell carcinoma treated with immune checkpoint inhibitors. Cancer Res Treat 54:505–516. 10.4143/crt.2020.1198 34176250 10.4143/crt.2020.1198 PMC9016310

[22] Facchinetti F, Mazzaschi G, Barbieri F et al (2020) First-line pembrolizumab in advanced non-small cell lung cancer patients with poor performance status. Eur J Cancer 130:155–167. 10.1016/j.ejca.2020.02.023 32220780 10.1016/j.ejca.2020.02.023

[23] Xu J, Bai Y, Li E, Xu N, Shi D, Qian J (2022) Efficacy and safety of chemotherapy regimens for first-line treatment of advanced esophageal squamous cell carcinoma in Asia: a systematic review. Expert Rev Anticancer Ther 22:981–998. 10.1080/14737140.2022.2110470 35950848 10.1080/14737140.2022.2110470

[24] Hu G, Wang Z, Wang Y, Zhang Q, Tang N, Guo J, Liu L, Han X (2016) Comparison of cisplatinum/paclitaxel with cisplatinum/5-fluorouracil as first-line therapy for nonsurgical locally advanced esophageal squamous cell carcinoma patients. Drug Des Devel Ther 10:2129–2136. 10.2147/dddt.S105441 27445460 10.2147/DDDT.S105441 PMC4936807

[25] Wang ZX, Cui C, Yao J et al (2022) Toripalimab plus chemotherapy in treatment-naïve, advanced esophageal squamous cell carcinoma (JUPITER-06): a multi-center phase 3 trial. Cancer Cell 40:277–88.e3. 10.1016/j.ccell.2022.02.007 35245446 10.1016/j.ccell.2022.02.007

[26] Lu Z, Wang J, Shu Y et al (2022) Sintilimab versus placebo in combination with chemotherapy as first line treatment for locally advanced or metastatic oesophageal squamous cell carcinoma (ORIENT-15): multicentre, randomised, double blind, phase 3 trial. BMJ 377:e068714. 10.1136/bmj-2021-068714 35440464 10.1136/bmj-2021-068714 PMC9016493

[27] Xu J, Kato K, Raymond E et al (2023) Tislelizumab plus chemotherapy versus placebo plus chemotherapy as first-line treatment for advanced or metastatic oesophageal squamous cell carcinoma (RATIONALE-306): a global, randomised, placebo-controlled, phase 3 study. Lancet Oncol 24:483–495. 10.1016/s1470-2045(23)00108-0 37080222 10.1016/S1470-2045(23)00108-0

[28] Liu Z, Liu J, Li Z (2022) Chemoimmunotherapy in advanced esophageal squamous cell carcinoma: optimizing chemotherapy regimens for immunotherapy combinations. Signal Transduct Target Ther 7:233. 10.1038/s41392-022-01077-w 35831276 10.1038/s41392-022-01077-w PMC9279420

[29] Li Y, Ji Y, Shen L, Yin X, Huang T, Deng B, Guo H, Wu Y, Chen Y (2022) Clinical efficacy of combination therapy of an immune checkpoint inhibitor with taxane plus platinum versus an immune checkpoint inhibitor with fluorouracil plus platinum in the first-line treatment of patients with locally advanced, metastatic, or recurrent esophageal squamous cell carcinoma. Front Oncol 12:1015302. 10.3389/fonc.2022.1015302 36605427 10.3389/fonc.2022.1015302 PMC9808083

[30] Zhang Y, Shen G, Xu R et al (2023) Effectiveness and safety of camrelizumab-containing neoadjuvant therapy in patients with esophageal squamous cell carcinoma: a prospective multicenter observational cohort study. J Thorac Dis. 15:6228–37. 10.21037/jtd-23-1408 38090323 10.21037/jtd-23-1408 PMC10713292

[31] Xu CR, Chen Q, Zhou C et al (2023) Effectiveness and safety of camrelizumab in inoperable or advanced non-small cell lung cancer patients: a multicenter real-world retrospective observational study (CTONG2004-ADV). Transl Lung Cancer Res. 12:127–40. 10.21037/tlcr-22-852 36762057 10.21037/tlcr-22-852 PMC9903092

[32] Wang R, Shi M, Ji M et al (2023) Real world experience with camrelizumab in patients with advanced non-small cell lung cancer: a prospective multicenter cohort study (NOAH-LC-101). Transl Lung Cancer Res. 12:786–96. 10.21037/tlcr-23-121 37197631 10.21037/tlcr-23-121 PMC10183390

[33] Zhang Z, Xie T, Qi C, Zhang X, Shen L, Peng Z (2022) Peripheral blood biomarkers predictive of efficacy outcome and immune-related adverse events in advanced gastrointestinal cancers treated with checkpoint inhibitors. Cancers (Basel). 10.3390/cancers14153736 35954401 10.3390/cancers14153736 PMC9367581

[34] Chen Y, Zhang C, Peng Z, Qi C, Gong J, Zhang X, Li J, Shen L (2021) Association of lymphocyte-to-monocyte ratio with survival in advanced gastric cancer patients treated with immune checkpoint inhibitor. Front Oncol 11:589022. 10.3389/fonc.2021.589022 34141607 10.3389/fonc.2021.589022 PMC8203902

[35] Wang L, Zhu Y, Zhang B, Wang X, Mo H, Jiao Y, Xu J, Huang J (2022) Prognostic and predictive impact of neutrophil-to-lymphocyte ratio and HLA-I genotyping in advanced esophageal squamous cell carcinoma patients receiving immune checkpoint inhibitor monotherapy. Thorac Cancer 13:1631–1641. 10.1111/1759-7714.14431 35437954 10.1111/1759-7714.14431 PMC9161342

[36] Yodying H, Matsuda A, Miyashita M, Matsumoto S, Sakurazawa N, Yamada M, Uchida E (2016) Prognostic significance of neutrophil-to-lymphocyte ratio and platelet-to-lymphocyte ratio in oncologic outcomes of esophageal cancer: a systematic review and meta-analysis. Ann Surg Oncol 23:646–654. 10.1245/s10434-015-4869-5 26416715 10.1245/s10434-015-4869-5

[37] Guven DC, Sahin TK, Erul E, Rizzo A, Ricci AD, Aksoy S, Yalcin S (2022) The association between albumin levels and survival in patients treated with immune checkpoint inhibitors: a systematic review and meta-analysis. Front Mol Biosci 9:1039121. 10.3389/fmolb.2022.1039121 36533070 10.3389/fmolb.2022.1039121 PMC9756377

[38] Takeda K, Umezawa R, Takahashi N, Matsushita H, Kozumi M, Ishikawa Y, Yamamoto T, Takeda K, Jingu K (2018) Impact of change in serum albumin level during and after chemoradiotherapy in patients with locally advanced esophageal cancer. Esophagus 15:190–197. 10.1007/s10388-018-0612-1 29951984 10.1007/s10388-018-0612-1

