# Supplementary material for: Effectiveness, safety, and patterns of use of camrelizumab in advanced esophageal cancer: an individual patient data pooled analysis of 987 patients from three prospective cohort studies
Source: Cancer Immunol Immunother. 2025 Mar 8;74(4):138. doi: 10.1007/s00262-025-03970-z (PMC11890476; doi:10.1007/s00262-025-03970-z)
Supplement: Supplementary file 1 — Supplementary file1 [file 262_2025_3970_MOESM1_ESM.pdf]

Supplementary data

Supplementary Figure 1 Geographical distribution of patients included in the study.

Supplementary Figure 2 Kaplan-Meier curves for time to treatment discontinuation.

Supplementary Figure 3 Subgroup analyses of progression-free survival (PFS) in patients receiving camrelizumab in the first-line setting.

Supplementary Figure 4 Subgroup analyses of overall survival (OS) in patients receiving camrelizumab in the first-line setting.

Supplementary Figure 5 Subgroup analyses of progression-free survival (PFS) in patients receiving camrelizumab in the second line or later setting.

Supplementary Figure 6 Subgroup analyses of overall survival (OS) in patients receiving camrelizumab in the second line or later setting.

Supplementary Figure 7 Progression-free survival (PFS) and overall survival (OS) among patients of different age groups (<65, 65-75, and ≥75 years) in the first-line setting.

Supplementary Figure 8 Progression-free survival (PFS) and overall survival (OS) among patients of different age groups (<65, 65-75, and ≥75 years) in the second-line or later setting.

Supplementary Figure 9 Progression-free survival (PFS) and overall survival (OS) among patients of different Eastern Cooperative Oncology Group performance status (0-1, and ≥2) in the first-line setting.

Supplementary Figure 10 Progression-free survival (PFS) and overall survival (OS) among patients of different Eastern Cooperative Oncology Group performance status (0-1, and ≥2) in the second-line or later setting.

Supplementary Figure 11 Progression-free survival (PFS) and overall survival (OS) in

patients receiving camrelizumab combined with taxanes plus platinum (TP)- versus fluorouracil plus platinum (FP)-based chemotherapy in the first-line setting.

Supplementary Figure 12 Progression-free survival (PFS) and overall survival (OS) in patients receiving camrelizumab monotherapy or combination therapy in the second-line or later setting.

Supplementary Table 1 Summary of study design

Supplementary Table 2 List of study sites

Supplementary Table 3 Adverse events occurring in ≥5% of patients.

Supplementary Table 4 Adverse events occurring in ≥5% of patients aged ≥75 years.

Supplementary Table 5 Adverse events occurring in ≥5% of patients with an ECOG PS of ≥2.

Supplementary Table 6 Peripheral blood parameters used in Cox regression analyses

Supplementary Table 7 Summary of effectiveness outcomes of camrelizumab for advanced esophageal cancer in this study and pivotal clinical trials.

Supplementary Table 8 Multiple multivariable Cox regression model for overall survival analysis

Supplementary Figure 1 Geographical distribution of patients included in the study.

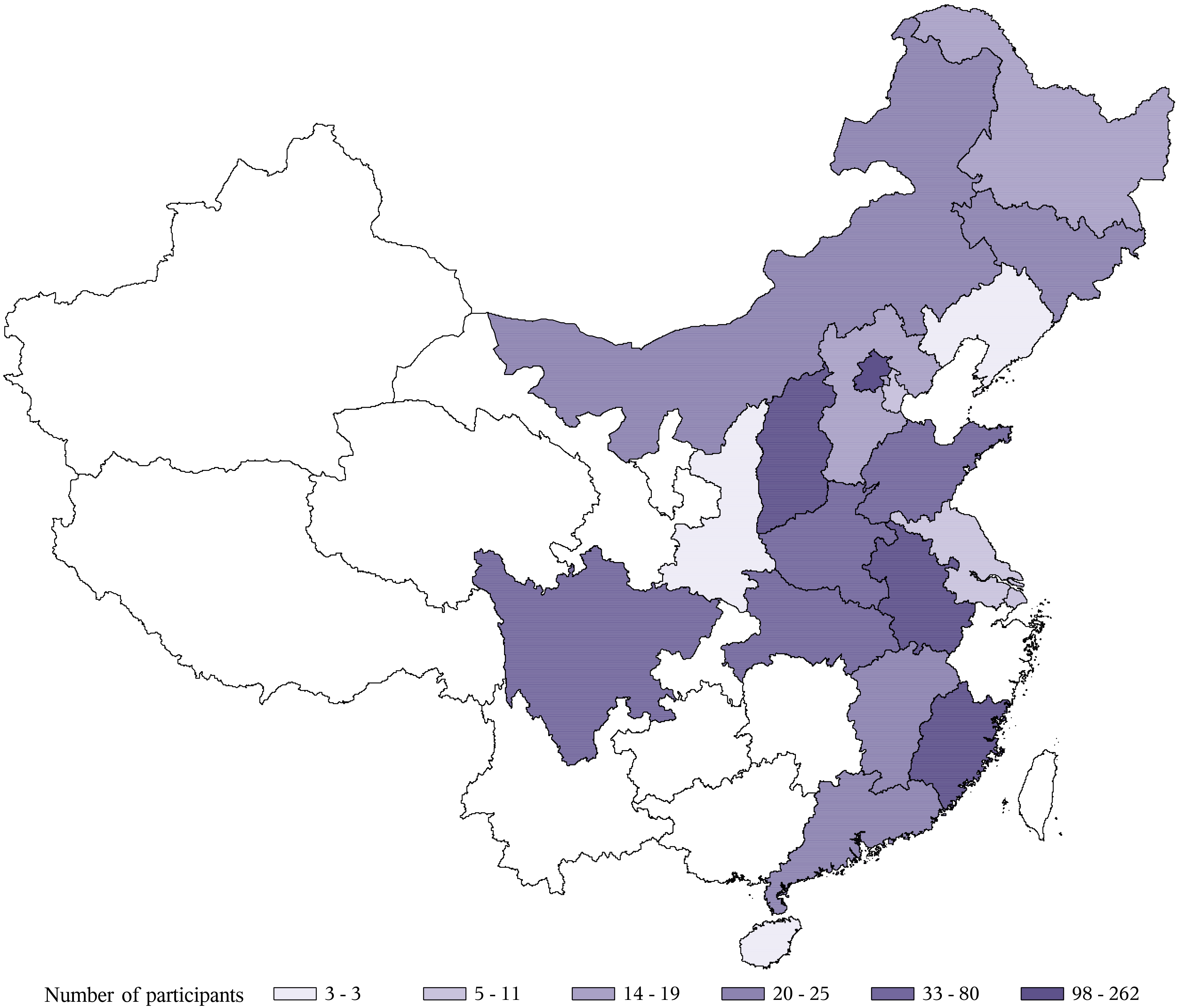

Supplementary Figure 2 Kaplan-Meier curves for time to treatment discontinuation.

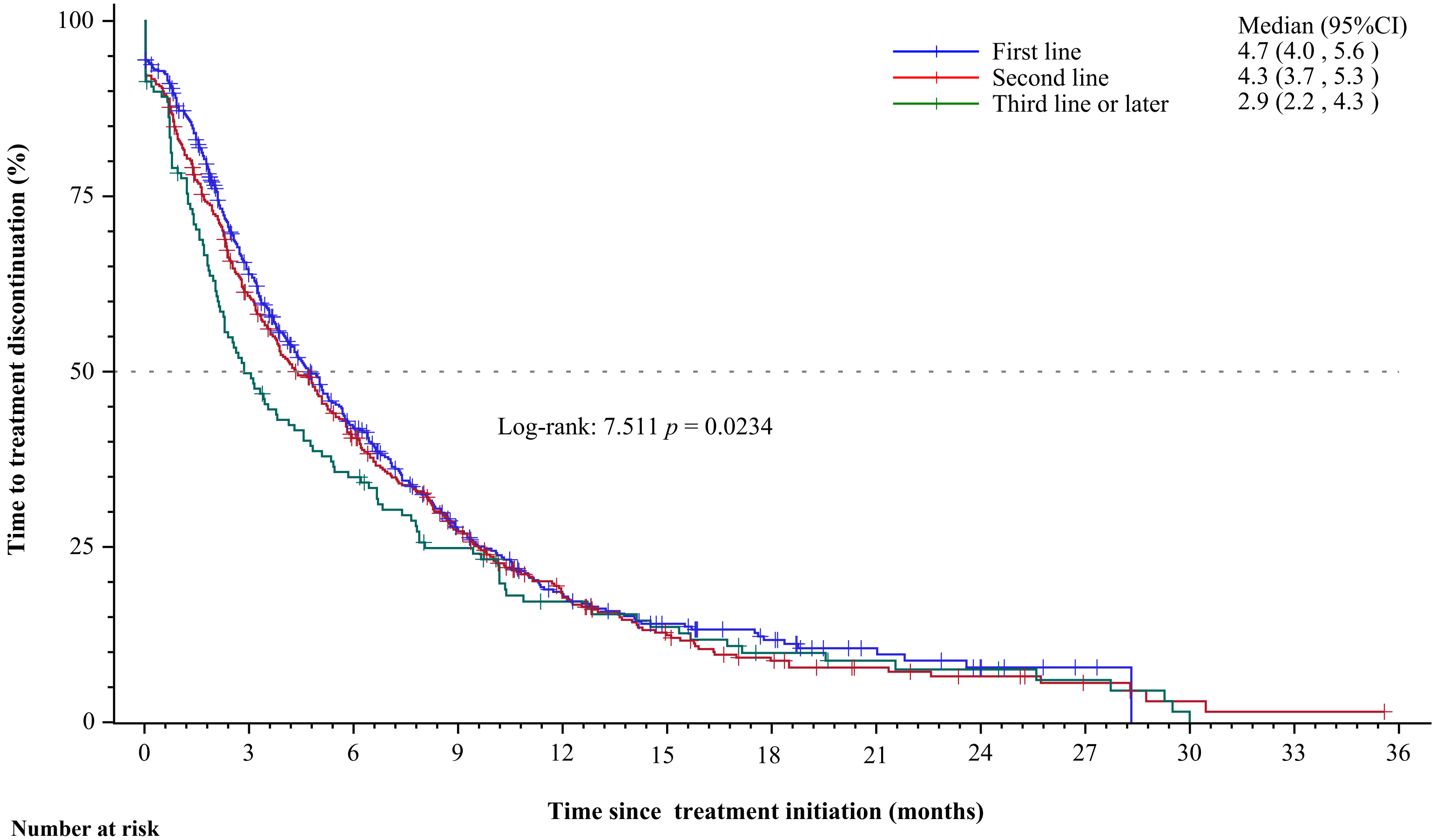

Number at risk

450	264	160	91	54	35	23	12	6	2	0		
398	232	147	93	53	33	20	13	9	5	2	1	0
139	68	47	31	19	15	9	7	6	4	0		

Supplementary Figure 3 Subgroup analyses of progression-free survival (PFS) in patients receiving camrelizumab in the first-line setting.

First-line	Events/N	Median PFS (95%CI)	12m PFS%	24m PFS%
**Total**	**129/450**	**9.9 (7. 4, 14. 4)**	**62.0 (55.4, 67.9)**	**47.5 (39.8, 54.9)**
Age, years				
<65	67/200	8.1 (5.7, 17. 0)	58.7 (49.1, 67.2)	40.8 (30.0, 51.2)
≥65, <75	48/183	13.5 (7.4, 15.5)	66.0 (55.0, 74.9)	51.4 (38.4, 63.0)
≥75	14/67	14.4 (5.3, NR)	63.9 (44.2, 78.2)	63.9 (44.2, 78.2)
ECOG PS				
0-1	113/387	9.2 (6.8, 14.7)	60.4 (53.3, 66.8)	48.1 (40.1, 55.7)
≥2	10/22	10.5 (3.2, 14.2)	64.3 (32.1, 84.2)	38.6 (10.3, 67.1)
Camrelizumab treatment patterns				
Monotherapy	14/31	3.7 (2.3, NR)	34.2 (13.0, 57.0)	34.2 (13.0, 57.0)
Plus chemotherapy (CT)	98/379	13.5 (7.8, 17.0)	65.8 (58.4, 72.1)	50.1 (41.5, 58.2)
Plus anti-angiogenic therapy (AT)	8/19	8.2 (2.0, NR)	50.3 (22.6, 72.8)	41.9 (16.3, 65.9)
Plus CT+AT	9/21	4.8 (2.6, NR)	48.4 (18.4, 73.3)	24.2 (1.6, 61.4)
CT regimens received				
Taxanes+ platinum (TP)	67/304	14.4 (7.8, 18.3)	69.9 (61.6, 76.8)	53.3 (42.9, 62.6)
Fluorouracil+ platinum	26/63	8.2 (4.7, 14.2)	52.4 (35.6, 66.7)	40.8 (23.9, 56.9)
TP regimens received				
Paclitaxel-based	29/122	8.3 (6.2, 17.0)	66.8 (52.0, 77.9)	47.5 (30.8, 62.4)
Nab-paclitaxel-based	32/156	NR (9.0, NR)	73.5 (62.3, 81.8)	59.2 (44.9, 70.9)
Docetaxel-based	6/26	8.6 (4.4, NR)	62.6 (30.0, 83.4)	NR (NR, NR)

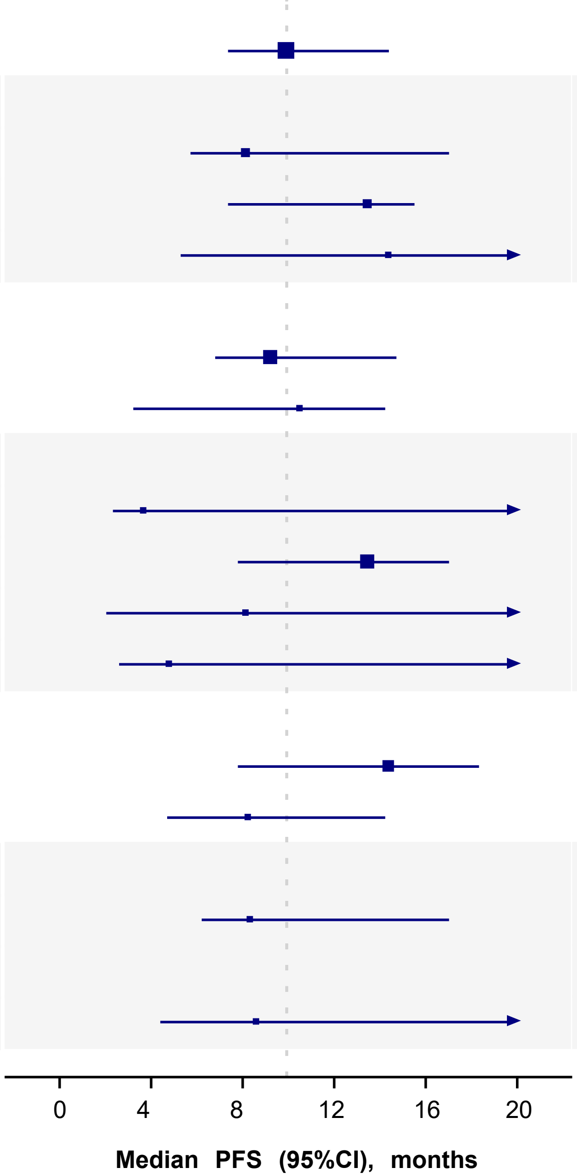

Supplementary Figure 4 Subgroup analyses of overall survival (OS) in patients receiving camrelizumab in the first-line setting.

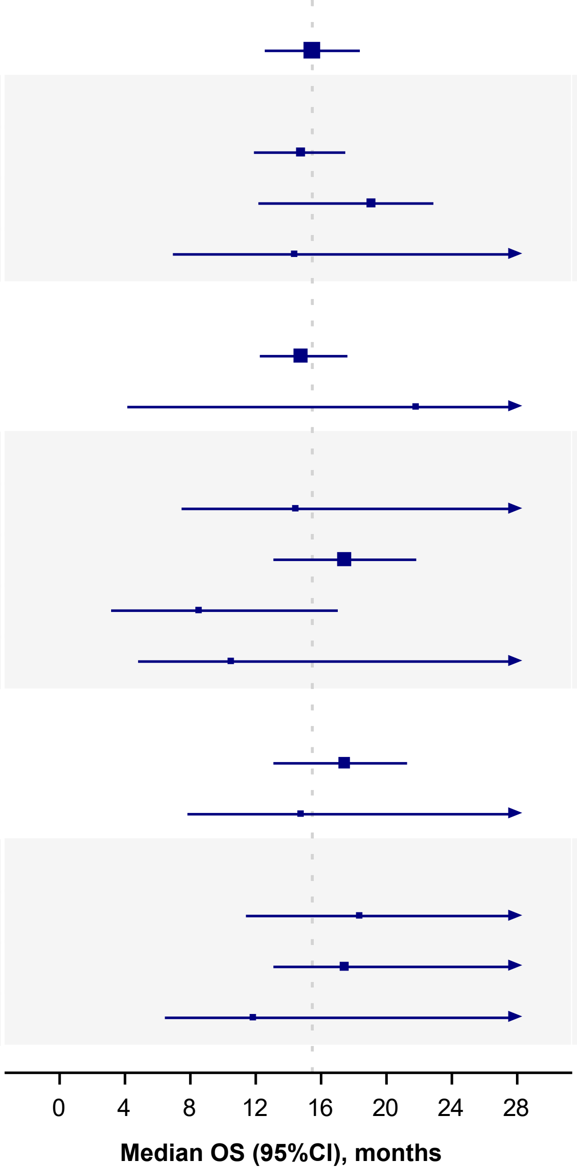

First-line	Events/N	MedianOS (95%CI)	12m OS%	24m OS%
**Total**	**142/450**	**15.5 (12. 6, 18. 4)**	**59.4 (52.9, 65.3)**	**32.5 (23.4, 42.0)**
Age, years				
<65	63/200	14.8 (11.9, 17 5)	59.6 (49.5, 68.4)	32.7 (20.9, 45.0)
≥65, <75	57/183	19.1 (12. 2, 22. 9)	61.6 (51.3, 70.3)	35.3 (21.7, 49.2)
≥75	22/67	14.4 (6.9, NR)	51.9 (34.1, 67.1)	37.1 (17.2, 57.2)
ECOG PS				
0-1	126/387	14.8 (12. 3, 17.6)	58.9 (51.9, 65.2)	34.2 (25.0, 43.6)
≥2	6/22	21.8 (4.1, NR)	64.2 (28.0, 85.7)	NR (NR, NR)
Camrelizumab treatment patterns				
Monotherapy	10/31	14.5 (7.5, NR)	57.3 (27.7, 78.5)	NR (NR, NR)
Plus chemotherapy (CT)	108/379	17.4 (13. 1, 21.8)	62.4 (55.1, 68.8)	35.5 (24.8, 46.3)
Plus anti-angiogenic therapy (AT)	13/19	8.5 (3. 2, 17.0)	42.7 (19.2, 64.4)	21.3 (5.5, 44.0)
Plus CT+AT	11/21	10.5 (4.8, NR)	40.5 (17.2, 62.8)	30.4 (9.2, 55.1)
CT regimens received				
Taxanes+ platinum (TP)	79/304	17.4 (13. 1, 21. 3)	63.5 (55.0, 70.8)	37.8 (26.0, 49.5)
Fluorouracil+ platinum	24/63	14.8 (7.8, NR)	55.6 (39.1, 69.3)	27.8 (9.0, 50.7)
TP regimens received				
Paclitaxel-based	27/122	18.4 (11.4, NR)	58.9 (42.4, 72.1)	NR (NR, NR)
Nab-paclitaxel-based	42/156	17.4 (13.1, NR)	69.4 (58.9, 77.8)	39.2 (24.3, 53.8)
Docetaxel-based	10/26	11.9 (6.4, NR)	43.3 (17.3, 67.0)	34.6 (11.4, 59.5)

Supplementary Figure 5 Subgroup analyses of progression-free survival (PFS) in patients receiving camrelizumab in the second line or later setting.

Second line or later	Events/N	Median PFS (95%CI)	12m OS%	24m OS%
**Total**	**230/537**	**6.2 (5. 3, 8.3)**	**51.0 (45.4, 56.3)**	**31.3 (25.4, 37.4)**
Age, years				
<65	123/281	5.6 (4.6, 8.1)	46.4 (38.6, 53.9)	30.4 (22.4, 38.7)
≥65, <75	85/192	7.8 (5 1, 9.6)	54.9 (45.7, 63.3)	33.1 (23.8, 42.8)
≥75	22/64	8.8 (3. 9, NR)	58.5 (40.8, 72.6)	28.2 (9.8, 50.1)
ECOG PS				
0-1	195/448	6.6 (5. 3, 8.8)	51.7 (45.7, 57.4)	32.6 (26.1, 39.1)
≥2	22/42	3.1 (1.6, 5.4)	28.0 (11.3, 47.6)	7.0 (0.5, 26.3)
Camrelizumab treatment patterns				
Monotherapy	28/80	10.0 (3.4, NR)	50.7 (35.8, 63.9)	46.1 (30.2, 60.7)
Plus chemotherapy (CT)	127/309	5.7 (4.9, 8. 3)	48.9 (41.1, 56.3)	28.4 (20.6, 36.7)
Plus anti-angiogenic therapy (AT)	58/104	6.0 (2.7, 9.1)	49.9 (38.7, 60.1)	27.2 (16.6, 38.9)
Plus CT+AT	17/44	10.0 (5.1, 19.1)	61.7 (40.4, 77.3)	39.3 (18.9, 59.2)
CT regimens received				
Taxanes+ platinum (TP)	62/177	8.3 (5. 3, 9.3)	53.9 (42.4, 64.1)	29.6 (18.5, 41.5)
Fluorouracil+ platinum	45/96	5.2 (3.9, 7. 7)	43.4 (30.6, 55.4)	30.2 (17.7, 43.8)
TP regimens received				
Paclitaxel-based	19/57	9.6 (4.6, 14.1)	58.4 (36.8, 74.9)	26.3 (7.4, 50.4)
Nab-paclitaxel-based	39/100	8.1 (4.8, 9. 1)	52.2 (37.4, 65.0)	28.9 (15.9, 43.2)
Docetaxel-based	4/20	NR (1.7, NR)	51.1 (13.8, 79.7)	51.1 (13.8, 79.7)

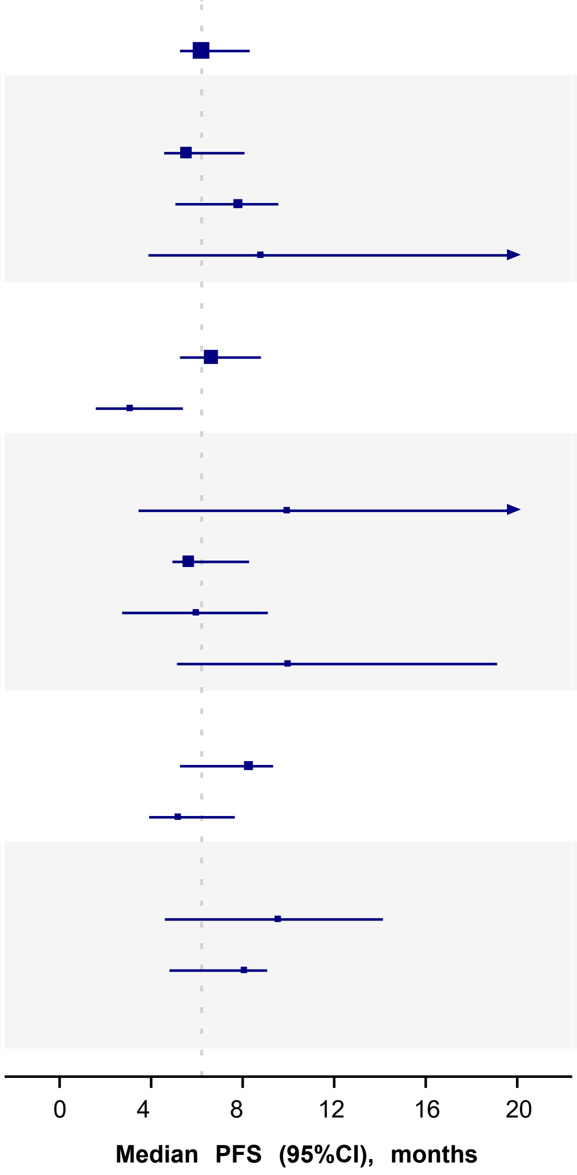

Supplementary Figure 6 Subgroup analyses of overall survival (OS) in patients receiving camrelizumab in the second line or later setting.

Second line or later	Events/N	Median OS (95%CI)	12m OS%	24m OS%
Total	**243/537**	**12.0 (10.0, 1 4.0)**	**49.8 (44.5, 54.8)**	**29.8 (23.6, 36.2)**
Age, years				
<65	131/281	12.2 (9.7, 1 5.0)	50.2 (43.1, 56.8)	29.4 (21.0, 38.3)
≥65, <75	91/192	10.7 (8.8, 13.6)	44.9 (35.7, 53.6)	24.1 (15.0, 34.4)
≥75	21/64	NR (9. 1, NR)	62.2 (46.8, 74.3)	50.8 (31.1, 67.5)
ECOG PS				
0-1	200/448	12.7 (10.6, 14.7)	52.1 (46.4, 57.5)	29.9 (23.0, 37.1)
≥2	27/42	4.1 (3. 1, 8.8)	23.0 (10.3, 38.6)	18.4 (6.9, 34.2)
Camrelizumab treatment patterns				
Monotherapy	32/80	27.7 (9 7, NR)	55.6 (42.3, 67.0)	50.4 (36.6, 62.7)
Plus chemotherapy (CT)	130/309	11.3 (9.7, 13.2)	47.6 (40.3, 54.6)	28.7 (19.6, 38.4)
Plus anti-angiogenic therapy (AT)	56/104	12.6 (8.8, 14.7)	50.9 (39.9, 60.9)	28.8 (17.4, 41.2)
Plus CT+AT	25/44	10.0 (6.7, 16 7)	44.7 (26.9, 61.0)	15.2 (4.5, 32.0)
CT regimens received				
Taxanes+ platinum (TP)	76/177	10.6 (8.7, 13.2)	43.9 (34.0, 53.4)	23.7 (13.8, 35.2)
Fluorouracil+ platinum	39/96	12.7 (8. 4, 25.7)	52.3 (39.0, 64.0)	31.9 (15.2, 50.0)
TP regimens received				
Paclitaxel-based	24/57	10.7 (7.7, 12.9)	41.4 (23.7, 58.3)	NR (NR, NR)
Nab-paclitaxel-based	40/100	11.7 (9 3, 16.7)	49.6 (36.3, 61.5)	28.7 (15.2, 43.8)
Docetaxel-based	12/20	7.6 (1.9, 11.0)	17.6 (1.4, 49.1)	NR (NR, NR)

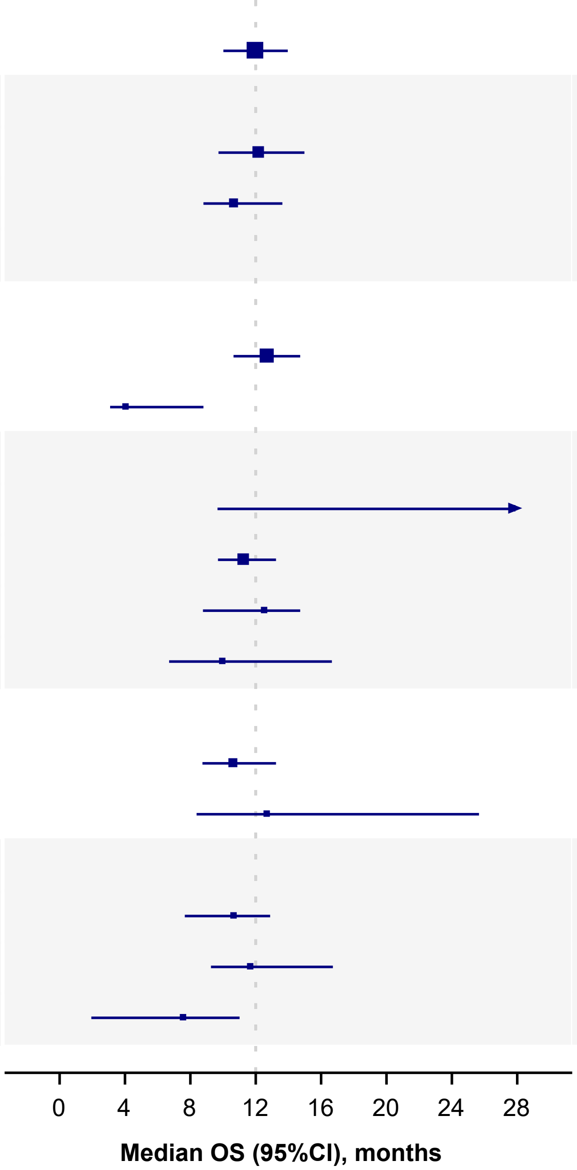

Supplementary Figure 7

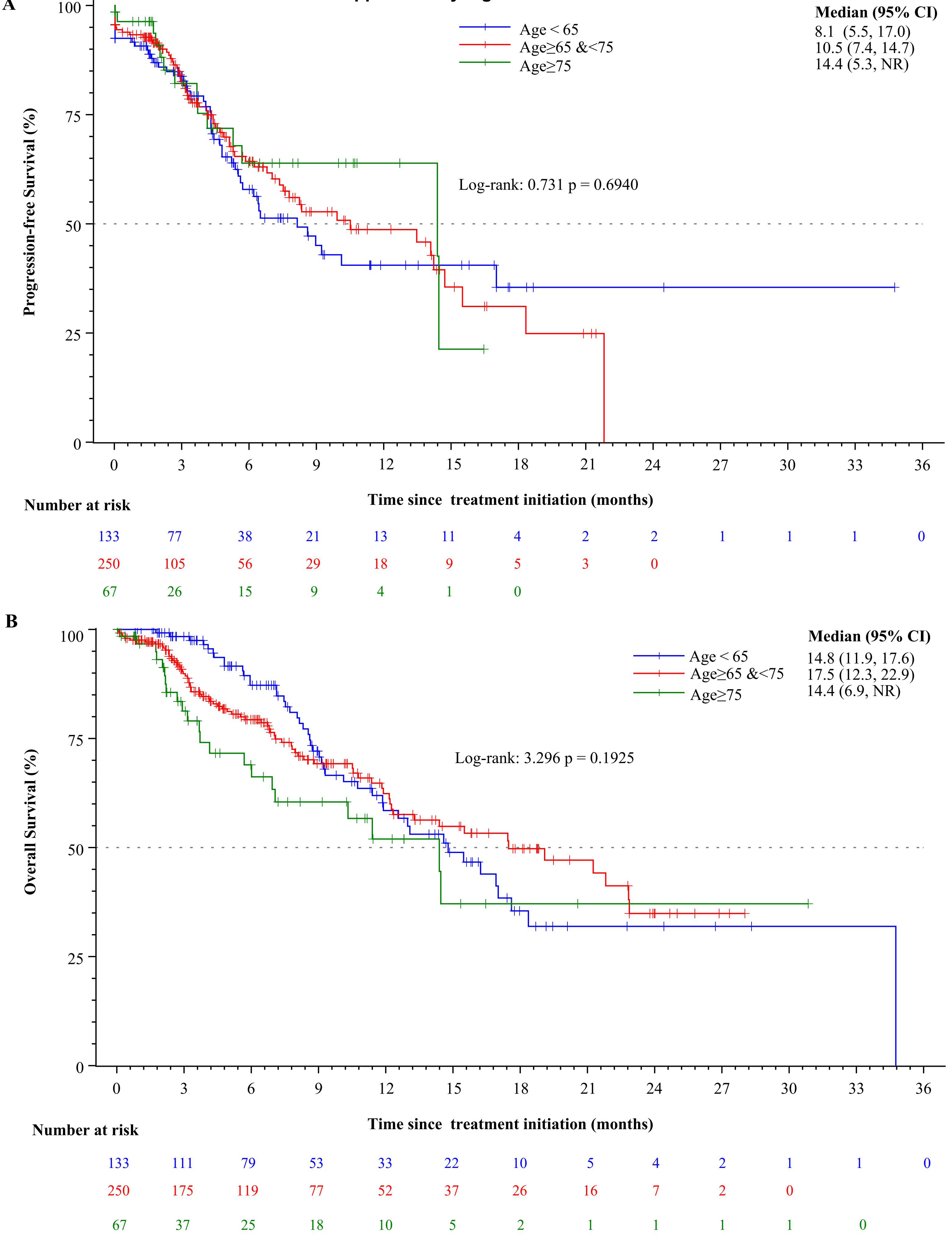

Supplementary Figure 8

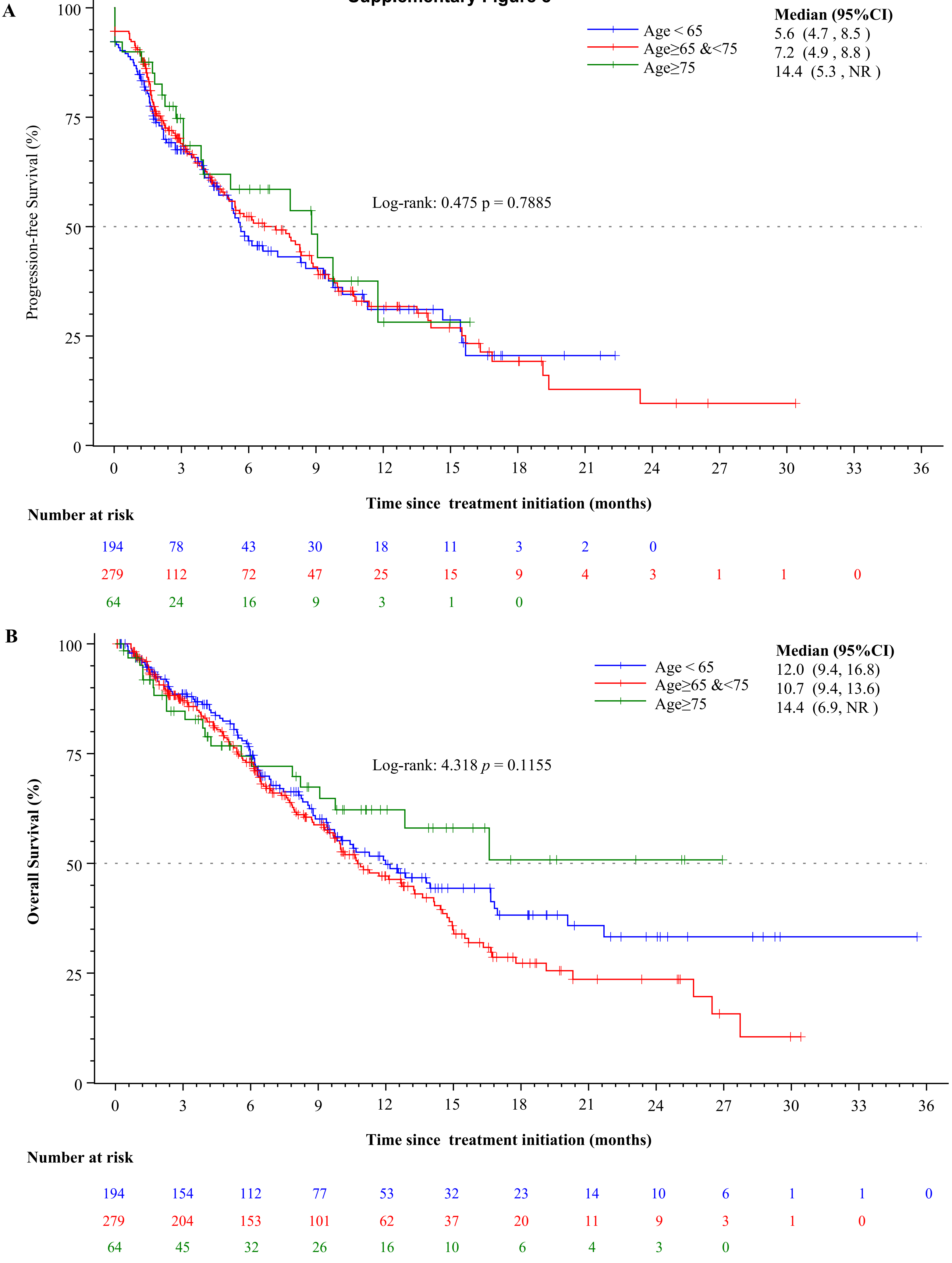

Supplementary Figure 9

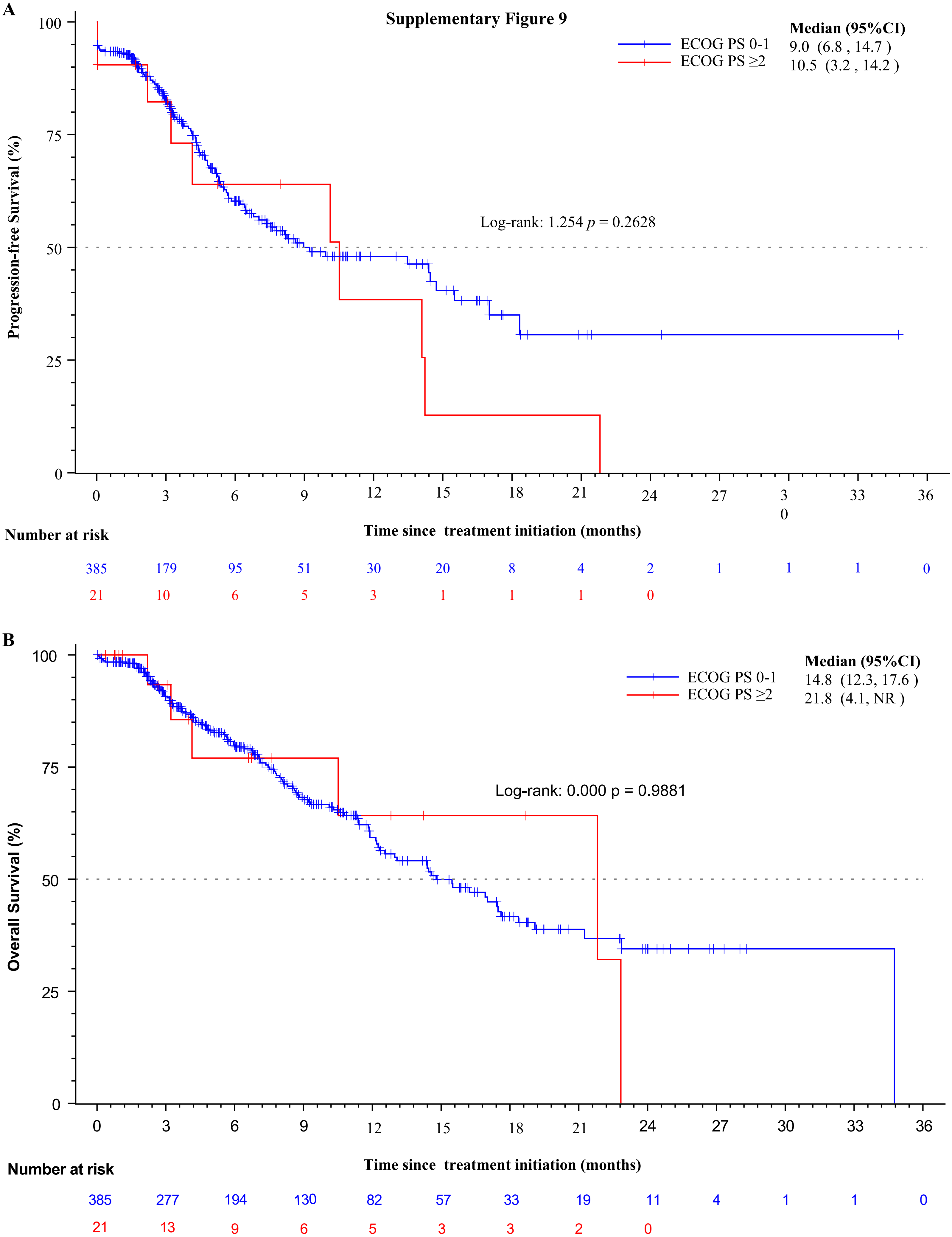

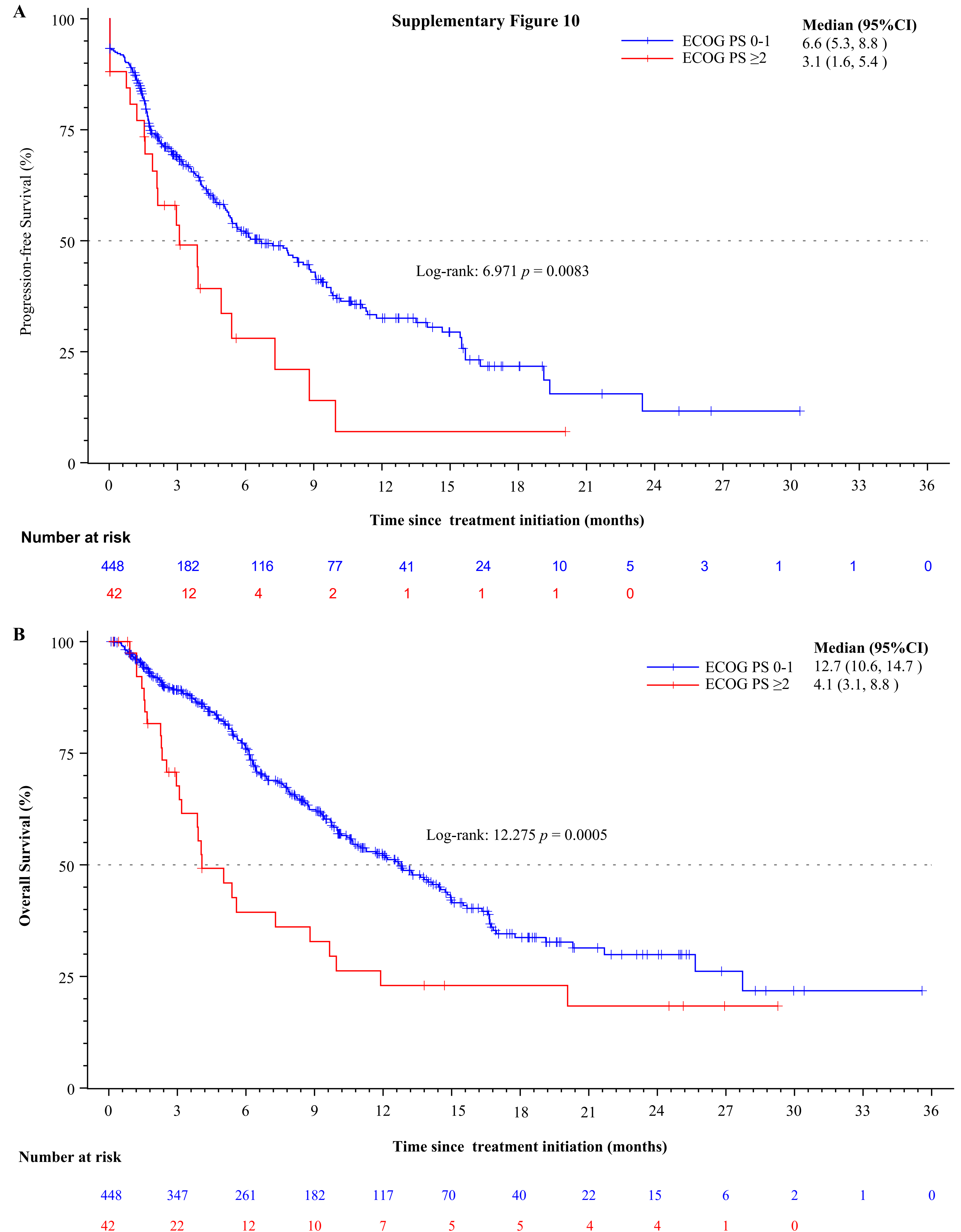

Supplementary Figure 10

Supplementary Figure 11

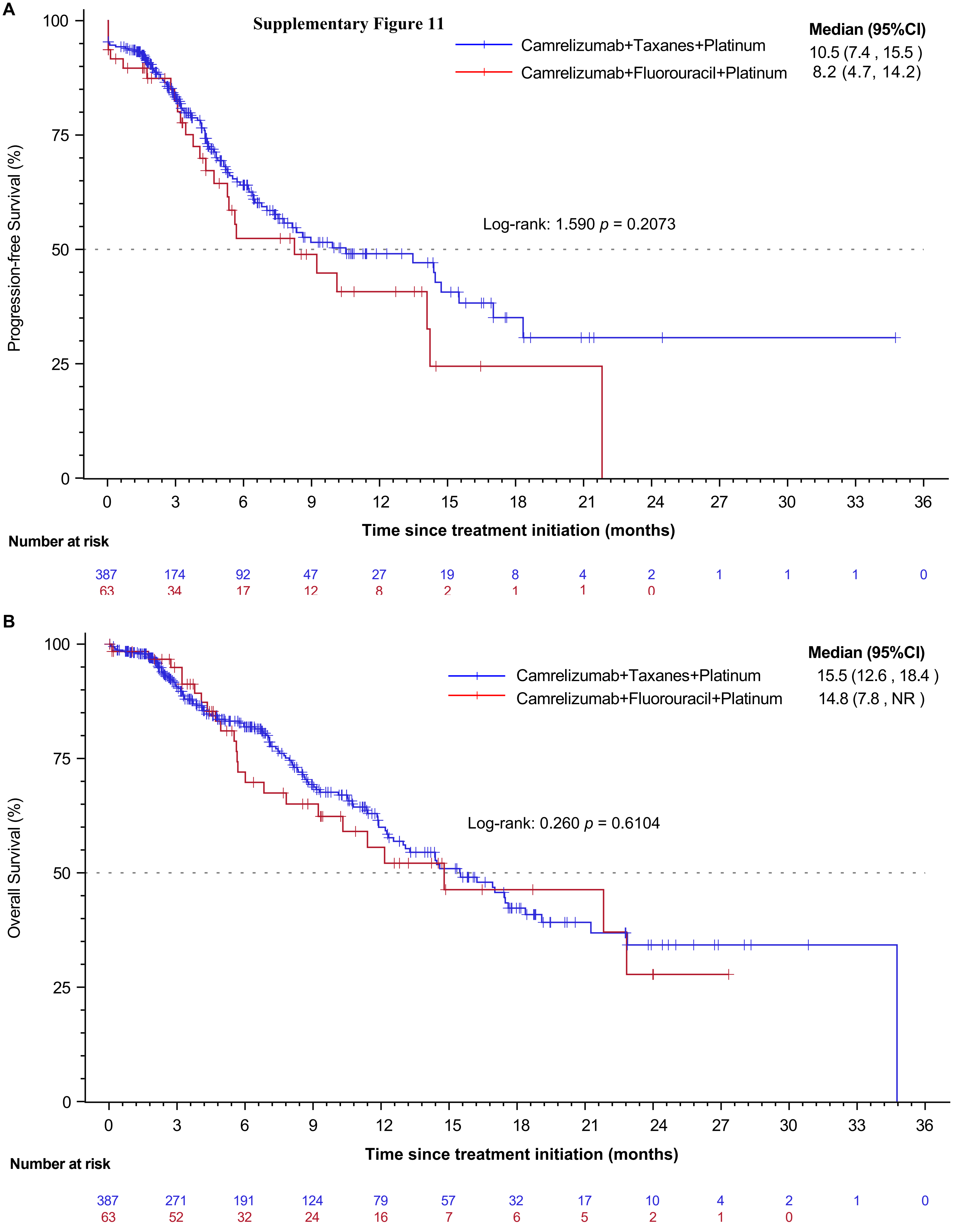

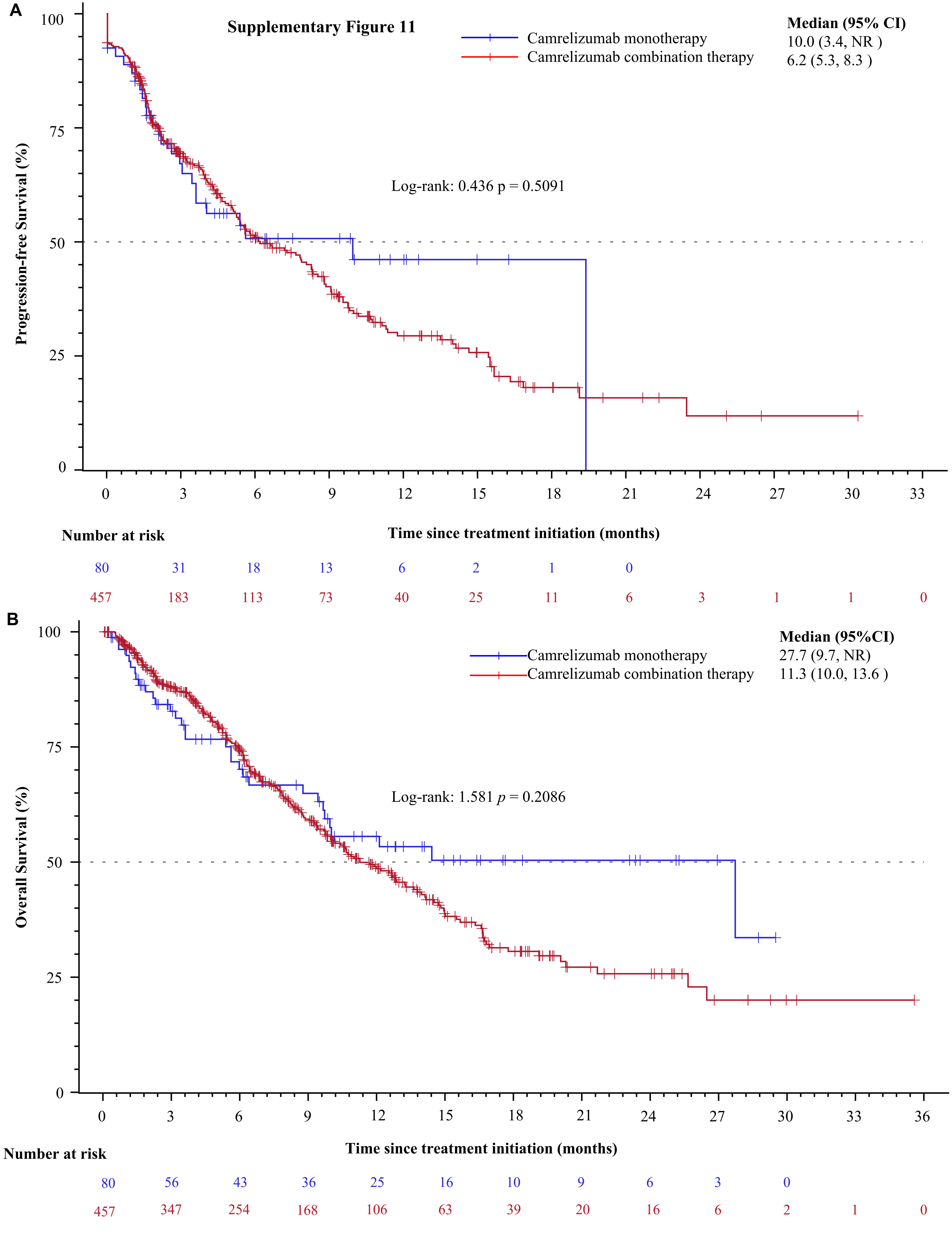
A
Supplementary Figure 11
Camrelizumab monotherapy
Camrelizumab combination therapy
Median (95% CI)
10.0 (3.4, NR)
6.2 (5.3, 8.3)
Log-rank: 0.436 p = 0.5091
Progression-free Survival (%)
100
75
50
25
0
0
3
6
9
12
15
18
21
24
27
30
33
Time since treatment initiation (months)
Number at risk
80 31 18 13 6 2 1 0
457 183 113 73 40 25 11 6 3 1 1 0
B
Median (95%CI)
Camrelizumab monotherapy
Camrelizumab combination therapy
27.7 (9.7, NR)
11.3 (10.0, 13.6)
Log-rank: 1.581 p = 0.2086
Overall Survival (%)
100
75
50
25
0
0
3
6
9
12
15
18
21
24
27
30
33
36
Time since treatment initiation (months)
Number at risk
80 56 43 36 25 16 10 9 6 3 0
457 347 254 168 106 63 39 20 16 6 2 1 0

Supplementary Table 1 Summary of study designs.

	**ESCORT-RWS/NCT04616040**	**NOAH-EC201/ChiCTR1900027275**	**ChiCTR2000039499**
Inclusion/exclusion criteria	1. Age ≥18 years<br>2. Histologically or cytologically confirmed locally advanced unresectable, recurrent or metastatic esophageal cancer<br>3. Eligible for camrelizumab at investigator's discretion	1. Age≥18 years<br>2. Histologically or cytologically confirmed esophageal cancer<br>3. At least one measurable lesion<br>4. ECOG PS of 0-2	1. Age ≥ 18 years<br>2. Pathologically confirmed esophageal cancer at clinical stage II-III<br>3. At most one prior systemic therapy<br>4. At least one measurable lesion per RECIST 1.1<br>5. ECOG PS of 0-1
Clinical settings	First, second, or third line of later	First, second, or third line of later	First or second line
Treatment	Camrelizumab monotherapy or in combination	Camrelizumab-containing regimens	Camrelizumab monotherapy or in combination
Radiologic assessment	Every 6~8 weeks	Every 3-4 months	Every 6 (±1) weeks
Follow-up	Every 3-6 months	Every 3-6 months	Every 3-6 months

Supplementary Table 2 List of study sites

No.	Study site	Geographic location	Hospital level	Patients
1	Peking University Cancer Hospital	Beijing	Tertiary	96
2	Changzhi People's Hospital Affiliated to Changzhi Medical College	Shanxi	Tertiary	68
3	Fujian Cancer Hospital, Fujian Medical University Cancer Hospital	Fujian	Tertiary	43
4	The First Affiliated Hospital of Bengbu Medical College	Anhui	Tertiary	40
5	The First Affiliated Hospital of USTC, Anhui Provincial Hospital	Anhui	Tertiary	38
6	Provincial Hospital Affiliated to Shandong First Medical University	Shandong	Tertiary	35
7	Suining Central Hospital	Sichuan	Tertiary	35
8	Anyang Tumor Hospital	Henan	Tertiary	33
9	Shandong Cancer Hospital and Institute	Shandong	Tertiary	30
10	Affiliated Cancer Hospital of Shanxi Medical University	Shanxi	Tertiary	30
11	Anhui Provincial Cancer Hospital	Anhui	Tertiary	29
12	The Second Affiliated Hospital of Anhui Medical University	Anhui	Tertiary	28
13	Hubei Cancer Hospital, Tongji Medical College, Huazhong University of Science and Technology	Hubei	Tertiary	21
14	The First Affiliated Hospital of Xinxiang Medical University	Henan	Tertiary	20
15	Yijishan Hospital of Wannan Medical College	Anhui	Tertiary	20
16	Harbin Medical University Cancer Hospital	Heilongjiang	Tertiary	19
17	The First Affiliated Hospital of Anhui Medical University	Anhui	Tertiary	19
18	Ganzhou People's Hospital	Jiangxi	Tertiary	18
19	Nanyang Central Hospital	Henan	Tertiary	17
20	Anxi County Hospital	Fujian	Tertiary	15
21	Cangzhou Central Hospital	Hebei	Tertiary	14
22	Sichuan Province Hospital	Sichuan	Tertiary	12
23	Union Hospital, Tongji Medical College, Huazhong University of Science and Technology	Hubei	Tertiary	12
24	The First Affiliated Hospital of Anhui University of Traditional Chinese Medicine	Anhui	Tertiary	12
25	Zhongshan Hospital Affiliated to Xiamen University	Fujian	Tertiary	12
26	Lixin County People's Hospital	Anhui	Tertiary	11
27	Zhangzhou Hospital Affiliated to Fujian Medical University	Fujian	Tertiary	11
28	China-Japan Union Hospital of Jilin University	Ji Lin	Tertiary	10
29	First Hospital of Jilin University	Ji Lin	Tertiary	10
30	Chifeng Cancer Hospital	Inner Mongolia	Tertiary	10
31	Affiliated Hospital of Chifeng University	Inner Mongolia	Tertiary	10
32	Cancer Hospital affiliated to Shantou University Medical College	Guangdong	Tertiary	9
33	Putian University Affiliated Hospital	Fujian	Tertiary	9
34	West China Hospital of Sichuan University	Sichuan	Tertiary	8
35	Tongling People's Hospital	Anhui	Tertiary	8
36	Guangdong Provincial Hospital of Traditional Chinese Medicine	Guangdong	Tertiary	7
37	The First Hospital of Putian	Fujian	Tertiary	7

38	The 900th Hospital of the Joint Logistics Support Force of the Chinese People's Liberation Army Putian Medical District	Fujian	Tertiary	7
39	Henan Provincial People's Hospital	Henan	Tertiary	6
40	Xin Hua Hospital Affiliated to Shanghai Jiao Tong University School of Medicine	Shanghai	Tertiary	6
41	The 901st Hospital of the Joint Logistics Support Force of the Chinese People's Liberation Army	Anhui	Tertiary	6
42	Quanzhou First Hospital Affiliated to Fujian Medical University	Fujian	Tertiary	6
43	Sun Yat-Sen Memorial Hospital, Sun Yat-Sen University	Guangdong	Tertiary	5
44	Jiangsu Province Hospital	Jiangsu	Tertiary	5
45	Tianjin Medical University Cancer Institute & Hospital	Tianjin	Tertiary	5
46	The Affiliated Hospital of Qingdao University	Shandong	Tertiary	5
47	Jinshan Hospital of Fudan University	Shanghai	Tertiary	5
48	Fuyang People's Hospital	Anhui	Tertiary	5
49	Huaibei People's Hospital	Anhui	Tertiary	5
50	Fexi People's Hospital	Anhui	Tertiary	5
51	The First Affiliated Hospital of Xiamen University	Fujian	Tertiary	5
52	The Third Affiliated Hospital, Sun Yat-Sen University	Guangdong	Secondary	4
53	The Second Affiliated Hospital of Soochow University	Jiangsu	Secondary	4
54	The Hospital of Suixi County	Anhui	Secondary	4
55	Wangjiang People's Hospital of Anhui	Anhui	Secondary	4
56	The Second Affiliated Hospital of Xiamen Medical College	Fujian	Tertiary	4
57	Fujian Medical University Union Hospital	Fujian	Secondary	4
58	Beijing Luhe Hospital, Capital Medical University	Beijing	Secondary	3
59	Henan Cancer Hospital, Affiliated Cancer Hospital of Zhengzhou University	Henan	Tertiary	3
60	The Second Affiliated Hospital of Xi'an Jiaotong University	Shaanxi	Tertiary	3
61	Hainan Cancer Hospital	Hainan	Secondary	3
62	Panjin central hospital	Liaoning	Tertiary	3
63	Xuancheng People's Hospital	Anhui	Tertiary	3
64	The First People's Hospital of Chuzhou	Anhui	Tertiary	3
65	The 910th Hospital of the Joint Logistics Support Force of the Chinese People's Liberation Army	Fujian	Secondary	3
66	The First Affiliated Hospital of Nanchang University	Jiangxi	Secondary	2
67	Beijing Daxing District People's Hospital	Beijing	Tertiary	2
68	Beijing Tsinghua Changgung Hospital	Beijing	Tertiary	2
69	Wan Bei General Hospital of Wanbei Coal Power Group	Anhui	Tertiary	2
70	Guangde Hospital of Traditional Chinese Medicine	Anhui	Tertiary	2
71	The Second People's Hospital of Huaiyuan	Anhui	Secondary	2
72	Huaibei Miner General Hospital	Anhui	Tertiary	2
73	Fengyang County People's Hospital	Anhui	Secondary	2
74	The Second Affiliated Hospital of Bengbu Medical College	Anhui	Tertiary	2
75	Nanping First Hospital, Fujian Medical University	Fujian	Tertiary	2
76	The Second Affiliated Hospital of Fujian Medical University	Fujian	Tertiary	2
77	The First Affiliated Hospital of Henan University	Henan	Tertiary	1

78	Fuyang Cancer Hospital	Anhui	Tertiary	1
79	Huaiyuan People's Hospital	Anhui	Tertiary	1
80	Shexian People's Hospital	Anhui	Tertiary	1
81	Guangde People's Hospital	Anhui	Tertiary	1
82	Fuyang Hospital of Anhui Medical University	Anhui	Tertiary	1
83	Suzhou Municipal Hospital	Anhui	Tertiary	1
84	Ma'anshan 17th Metallurgical Hospital	Anhui	Tertiary	1
85	The people's hospital of Susong	Anhui	Tertiary	1
86	The second People's Hospital of Anhui	Anhui	Tertiary	1
87	Huoqiu First People's Hospital	Anhui	Tertiary	1
88	The 909th Hospital of the Joint Logistics Support Force of the Chinese People's Liberation Army	Fujian	Tertiary	1
89	Mindong Hospital of Ningde City	Fujian	Tertiary	1
90	Longyan Second Hospital	Fujian	Tertiary	1
91	Sanming First Hospital	Fujian	Secondary	1

Supplementary Table 3 Adverse events occurring in ≥5% of patients.

	Total (n=987)
**Any adverse events, n (%)**	735 (74.5)
Anemia	295 (29.9)
White blood cells decreased	275 (27.9)
Neutrophil count decreased	193 (19.6)
Platelet count decreased	158 (16.0)
Nausea	128 (13.0)
Lymphocyte count decreased	107 (10.8)
Reactive cutaneous capillary endothelial proliferation	98 (9.9)
Vomiting	86 (8.7)
Weight loss	70 (7.1)
Fatigue	69 (7.0)
Decreased appetite	65 (6.6)
Cough	64 (6.5)
COVID-19 infection	64 (6.5)
Infectious pneumonia	63 (6.4)
Hypoalbuminemia	61 (6.2)
Hypokalemia	58 (5.9)
Hypoproteinemia	50 (5.1)
Aspartate aminotransferase increased	50 (5.1)

Supplementary Table 4 Adverse events occurring in ≥5% of patients aged ≥75 years.

	**Age≥75 years (n=131)**
**Any adverse events, n (%)**	91 (69.5)
Anemia	27 (20.6)
White blood cells decreased	21 (16.0)
Nausea	15 (11.5)
Reactive cutaneous capillary endothelial proliferation	14 (10.7)
Platelet count decreased	14 (10.7)
Lymphocyte count decreased	13 (9.9)
Neutrophil count decreased	13 (9.9)
Fever	12 (9.2)
Cough	11 (8.4)
Infectious pneumonia	9 (6.9)
Vomiting	9 (6.9)
Weight loss	9 (6.9)
Covid-19 infection	9 (6.9)
Fatigue	8 (6.1)
Hypokalemia	7 (5.3)

Supplementary Table 5 Adverse events occurring in ≥5% of patients with an ECOG PS of ≥2.

	**ECOG PS≥2 (n=63)**
**Any adverse events, n (%)**	44 (69.8)
Anemia	19 (30.2)
White blood cells decreased	10 (15.9)
Vomiting	10 (15.9)
Nausea	7 (11.1)
Cough	7 (11.1)
Lymphocyte count decreased	7 (11.1)
Infectious pneumonia	6 (9.5)
Neutrophil count decreased	6 (9.5)
Dysphagia	5 (7.9)
Diarrhea	4 (6.3)
Productive cough	4 (6.3)
Pleural effusion	4 (6.3)

Supplementary Table 6 Peripheral blood parameters used in Cox regression analyses

Variables	Full name	Normal value or range	Unit
ALB	Albumin	≥35	g/L
ALP	Alkaline phosphatase	40~160	U/L
ALT	Alanine aminotransferase	0~40	U/L
AMS	Amylase	0~110	IU/L
ANC	Absolute neutrophil count	2~8	10^9/L
AST	Aspartate aminotransferase	0~45	U/L
CEA	Carcinoembryonic antigen	<5	ng/ml
Cr	Creatinine	50~130	umol/L
DBIL	Direct bilirubin	0~6	umol/L
EOSO	Eosinophils	0.05 ~ 0.5	10^9/L
GGT	Gamma-glutamyltransferase	10~50	IU/L
GLU	Glucose	3.6~6.1	mmol/L
Hb	Hemoglobin	≥90	g/L
HDL	High-density lipoprotein	0.7~2.0	mmol/L
LDH	Lactate dehydrogenase	<240	U/L
LDL	Low-density lipoprotein	≤3.37	mmol/L
LMR	Lymphocyte-to-monocyte ratio	≥3.5	ratio
LY	Lymphocyte	1~5	10^9/L
MONO	Monocyte	0.12 ~ 0.8	10^9/L
NLR	Neutrophil to lymphocyte ratio	<3	ratio
PLR	Platelet to lymphocyte ratio	<173.7	ratio
PLT	Platelet count	100~350	10^9/L
RBC	Red blood cells	3.5~5.5	10^{12}/L
TBIL	Total bilirubin	1.7~20	umol/L
TP	Total protein	60~80	g/L
TSH	Thyroid-stimulating hormone	2~10	mU/L
WBC	White blood cells	4~10	10^9/L

Supplementary Table 7 Multiple multivariable Cox regression model for overall survival analysis

Explanatory variables (normal vs abnormal)	HR (95% CI)	*p* value*	FDR adjusted *p* value
LMR#	0.22 (0.09,0.50)	**<0.001**	**0.011**
HDL	1.27 (0.51,3.91)	0.643	0.821
RBC	0.72 (0.53,1.00)	**0.043**	0.194
ALB	0.54 (0.38,0.78)	**<0.001**	**0.011**
PLT	0.67 (0.47,0.97)	**0.028**	0.150
PLR#	0.36 (0.18,0.67)	**0.003**	**0.023**
CEA	0.76 (0.52,1.12)	0.147	0.320
AST	0.82 (0.49,1.46)	0.469	0.636
Mono	0.73 (0.50,1.11)	0.122	0.320
ALT	0.71 (0.45,1.20)	0.173	0.330
LDH	0.70 (0.47,1.07)	0.092	0.320
TP	0.75 (0.52,1.11)	0.136	0.320
NLR	0.65 (0.48,0.88)	**0.006**	**0.039**
Cr#	0.54 (0.26,1.22)	0.113	0.320
LDL	1.43 (0.86,2.50)	0.184	0.330
GGT	0.82 (0.55,1.23)	0.320	0.541
LY	0.81 (0.61,1.08)	0.154	0.320
DBIL	1.11 (0.71,1.81)	0.669	0.821
ALP	0.97 (0.54,1.94)	0.931	0.963
ANC	0.93 (0.65,1.37)	0.700	0.821
TSH#	0.53 (0.22,1.19)	0.139	0.320
Hb	1.05 (0.60,2.04)	0.864	0.963
AMS	2.04 (0.52,13.98)	0.374	0.594
EOSO	1.13 (0.85,1.52)	0.407	0.611
WBC	0.99 (0.75,1.31)	0.963	0.963
TBIL	0.99 (0.59,1.79)	0.959	0.963
GLU	1.15 (0.79,1.72)	0.471	0.636

Note: HR, Hazard ratios; ECOG PS, Eastern Cooperative Oncology Group performance status; LMR, Lymphocyte to monocyte ratio; HDL, High-density lipoprotein; RBC: Red blood cells; ALB, Albumin; PLT, Platelet count; PLR, Platelet to lymphocyte ratio; CEA, Carcinoembryonic antigen; AST, Aspartate aminotransferase; MONO, Monocyte; ALT, Alanine aminotransferase; LDH, Lactate dehydrogenase; TP, Total protein; NLR, Neutrophil to lymphocyte ratio; Cr, Creatinine; LDL, Low-density lipoprotein; GGT, Gamma-glutamyltransferase; LY, Lymphocyte; DBIL, Direct bilirubin; ALP, Alkaline phosphatase; ANC, Absolute neutrophil count; TSH, Thyroid stimulating hormone; Hb, Hemoglobin; AMS, Amylase; EOSO, Eosinophils; WBC, White blood cells; TBIL, Total bilirubin; GLU, Glucose.

Adjusted for baseline patient characteristics (age, sex, smoking status, drinking status, primary tumor location, liver metastases, lung metastases, bone metastases, line of therapy, and camrelizumab treatment patterns). #Proportional hazards assumption is not met in the raw Cox model, and time-dependent covariate CovT=Cov(log(time)) is

introduced in the final Cox model.

Supplementary Table 8 Summary of effectiveness outcomes of camrelizumab for advanced esophageal cancer in this study and pivotal clinical trials

	**Pooled analysis*#**			**ESCORT-1st**	**Zhang et al. 2020***	**ESCORT**	**CAP 02**
	**1st line (n=450)**	**2nd line (n=398)**	**3rd line (n=139)**	**1st line (n=298)**	**1st line (n=30)**	**2nd line (n=228)**	**2nd line (n=52)**
Treatment patterns	Cam/Cam+	Cam/Cam+	Cam/Cam+	Cam+TP	Cam+TP+Apa	Cam	Cam+Apa
Median follow-up (IQR), mons	8.7 (7.1, 9.6)	12.0 (10.4, 12.9)	11.7 (9.1, 13.8)	10.8 (7.3, 14.3)	25.0 (23.1, 26.2)	8.3 (4.1, 12.8)	7.5 (4.0, 11.2)
Median OS (95% CI), mons	15.5 (12.6, 18.4)	12.1 (10.0, 14.7)	10.9 (8.1, 14.5)	15.3 (12.8, 17.3)	19.4 (9.9, NR)	8.3 (6.8, 9.7)	15.8 (8.4, 16.2)
Median PFS (95% CI), mons	9.9 (7.4, 14.4)	6.6 (5.1, 8.8)	5.7 (3.1, 9.6)	6.9 (5.8, 7.4)	6.9 (4.5, 14.2)	1.9 (1.9, 2.4)	6.8 (3.8, 10.4)
ORR (95% CI)	45.5 (40.0, 51.0)	29.0 (23.9, 34.5)	22.9 (15.0, 32.6)	72.1 (66.7, 77.2)	80.0 (61.4, 92.3)	20.2 (15.2, 26.0)	34.6 (22.0, 49.1)
DCR (95% CI)	90.1 (86.3, 93.1)	79.5 (74.4, 83.9)	68.8 (58.5, 77.8)	91.3 (87.5, 94.2)	96.7 (82.8, 99.9)	44.7 (38.2, 51.4)	78.8 (65·3, 88·9)

Note: *Median follow-up is expressed as median and 95% confidence interval. #ORR and DCR are analyzed in the EAS population, otherwise in the ITT population. Cam, Camrelizumab monotherapy; Cam+: Camrelizumab in combination with other agents; TP, Paclitaxel and cisplatin; Apa, Apatinib; ITT, Intention to treat population; EAS. Effectiveness analysis set. IQR, Interquartile range; 95% CI, Confidence interval; ORR, Objective response rate; DCR, Disease control rate; PFS, Progression-free survival; OS, Overall survival.
